# Mechanical and Covalent Tailoring of Copper Catenanes for Selective Aqueous Nitrate-to-Ammonia Electrocatalysis

**DOI:** 10.1021/jacs.4c18547

**Published:** 2025-04-22

**Authors:** Yulin Deng, Xiaoyong Mo, Samuel Kin-Man Lai, Shu-Chih Haw, Ho Yu Au-Yeung, Edmund C. M. Tse

**Affiliations:** † HKU-CAS Joint Laboratory on New Materials & Department of Chemistry, The University of Hong Kong, Pokfulam Road, Hong Kong SAR, P. R. China; ‡ National Synchrotron Radiation Research Center, 101 Hsin-Ann Road, Hsinchu 30076, Taiwan; § State Key Laboratory of Synthetic Chemistry, The University of Hong Kong, Pokfulam Road, Hong Kong SAR, P. R. China

## Abstract

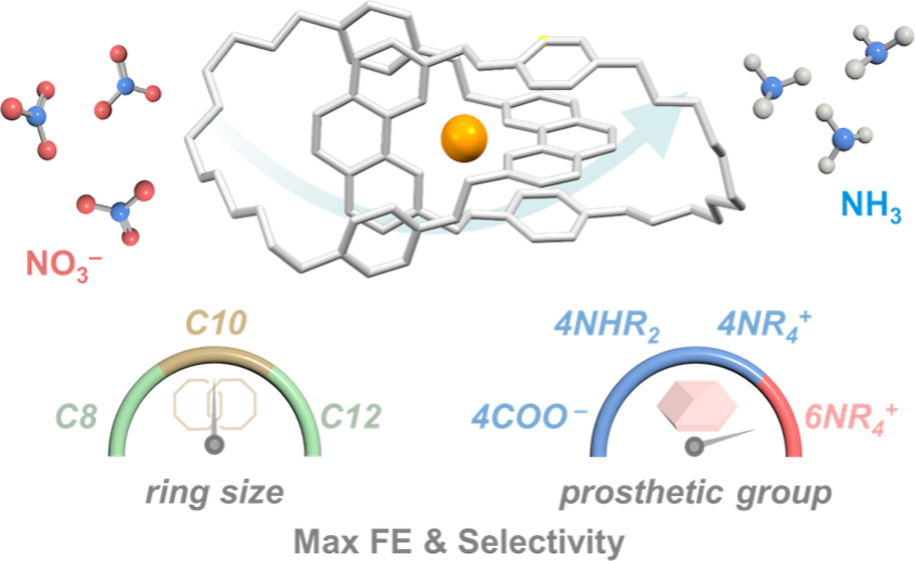

Electrocatalytic nitrate reduction reaction (NO_3_RR) for the selective generation of ammonia (NH_3_) enables the removal of deleterious nitrate pollutants while simultaneously upcycling them into a value-added fertilizer. The development of nonprecious metal-derived catalysts such as those featuring copper (Cu) as earth-abundant alternatives for the state-of-the-art precious metal catalysts is of urgent need yet suffering from the activity–selectivity–durability trilemma. Rational design of molecular Cu complexes with well-defined coordination structures permitting systematic structure–activity relationship (SAR) investigations is key to addressing the challenge. Here, a series of molecular Cu(I) complexes with [2]catenane ligands are developed as NO_3_RR electrocatalysts for the first time. By engineering multiple cationic ammoniums on the catenane backbone, acceptance of the anionic nitrate substrate as well as the release of the cationic ammonium product are promoted, thereby facilitating a higher Faradaic efficiency and product selectivity toward ammonia via an 8e^–^ pathway. Of note, the mutual Coulombic repulsion between the multiply charged ligands is overcome by the mechanical interlocking such that the catalyst integrity can be maintained under practical conditions. This report highlights the promise of employing mechanically interlocked ligands as a platform for customizing metal complexes as catalysts for redox processes involving multiple proton-coupled electron transfer steps.

## Introduction

Closing the artificial nitrogen cycle is the key to a sustainable society. The efficient removal of excessive nitrate (NO_3_^–^) and other nitrogen-containing pollutants from effluents, as well as their simultaneous upcycling into a value-added fertilizer (e.g., ammonia, NH_3_), is one central technology for achieving this goal.^1−3^ The efficient and selective electrocatalytic nitrate-to-ammonia conversion offers the opportunity for further generation of value-added nitrogen-containing organic compounds (NOCs) by harnessing ammonia from nitrate waste, including the production of ammonium carboxylates via reaction with carboxylic acids, the synthesis of urea in the presence of CO_2_ or carbonates, and metal-catalyzed amination in organic synthesis. In contrast to some common strategies for nitrate removal such as membrane technology, reverse osmosis, electrodialysis, and biological denitrification,^4−10^ electrocatalytic nitrate reduction is an environmentally friendly alternative that can remove and convert nitrate into ammonia via consecutive proton-coupled electron transfer (PCET) (NO_3_^–^ + 9H^+^ + 8e^–^ → NH_3_ + 3H_2_) under ambient conditions without additional oxygenation processes.^11−15^ Although the low-temperature nitrate-to-ammonia conversion method is promising, developing catalysts with high activity and selectivity toward ammonia remains challenging due to (1) the sluggish reduction kinetics, (2) multiple parallel pathways generating a mixture of products, and (3) the competing hydrogen evolution reaction (HER) at the cathode.^16−20^

Currently, clusters, nanoalloys, and nanoparticles derived from precious metals such as Pt, Pd, Ru, and Rh remain the major class of efficient and selective electrocatalysts for the nitrate reduction reaction (NO_3_RR), in which Faradaic efficiency (FE) can reach as high as 100%.^21−24^ Yet, the high cost of these precious metals has prohibited their practical application, and nonprecious metal (NPM) catalysts as cost-effective and scalable alternatives for NO_3_RR have attracted immense research interest.^25^ Copper (Cu) is particularly attractive due to its low cost, earth abundance, strong nitrate adsorption capability, and fast electron transfer kinetics. While Cu-based catalysts such as nanoalloys are promising for NO_3_RR,^26−33^ the activity–selectivity–durability trilemma remains hard to overcome due to the limitations in the precise, atomic-level tuning of the active site structure in these catalysts composed of bulk and nanoscale copper.^34,35^

In contrast, molecular transition metal complexes are amenable for structure–activity relationship (SAR) investigations via systematic, rational ligand design to allow for more precise control and tuning of the catalytic performance. Inspired by the active site structure of copper nitrite reductases, for example, Cu(II)-bis(pyridyl)amine complexes have been utilized for the selective reduction of nitrite (NO_2_^–^) into NO, and the relationship between the primary coordination structure and catalytic performance has been revealed by SAR studies using various ligand analogues.^36^ Copper(II) phthalocyanines have also been shown to enable NO_3_RR.^37−39^ Yet, different from the deeply buried metal active sites in metalloenzymes,^40−43^ molecular copper complexes are usually labile, and the rapid ligand exchange involving Cu^+^ or Cu^2+^ ions could often generate coordinatively ill-defined copper species during the catalysis,^44,45^ which not only complicate the SAR and mechanistic understanding of the NO_3_RR, but also the activity, selectivity, and durability of the catalyst are compromised.

Contrary to covalent modifications, mechanical interlocking is a conceptually different but effective way to circumvent issues related to labile copper exchange.^46−49^ Due to the interlocking, ligand dissociation and exchange are sufficiently inhibited, and the coordinated metal is kinetically stabilized.^50−55^ A well-defined coordination environment is thus maintained for sustaining the catalyst lifetime, minimizing side-reactivity, and extending the catalyst durability, despite there being a continuous change in the oxidation state, coordination number, and geometry of the copper during the catalysis.^55,56^ On the other hand, the dynamic coordination could also control substrate access, orient substrate in a specific direction, promote product elimination, and facilitate H^+^/e^–^ transfer for faster turnover, thereby enhancing the overall activity and selectivity.^57,58^

**Figure 1:**
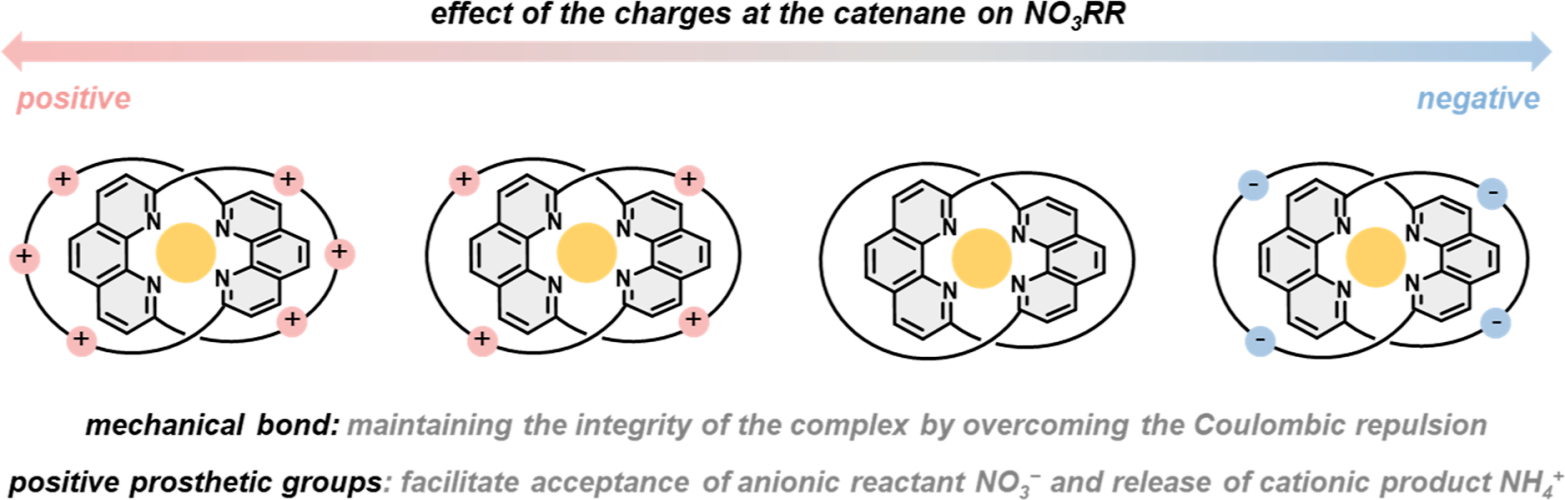
Effect of prosthetic groups of the Cu(I) catenane complexes on electrocatalytic nitrate reduction.

Herein, we report a study of enhancing the activity and selectivity of molecular Cu(I) catalysts for electrocatalytic NO_3_RR via the incorporation of electrically charged prosthetic groups on catenane ligands. While dissociation of the positively charged, mutually repelling ligands is prohibited by the mechanical interlocking and structural integrity of the molecular catalysts is maintained, acceptance of the negatively charged substrate (NO_3_^–^), as well as release of the positively charged product (NH_4_^+^), is also facilitated, resulting in promoted catalytic activity and product selectivity (Figure 1).^59^ In addition, the size of the interlocked macrocycles in the entangled ligands is also shown to influence the catalytic activity and selectivity of the Cu(I) catenanes.^56,57,60^

## Results and Discussion

### Catalyst Design and Synthesis

**Figure 2:**
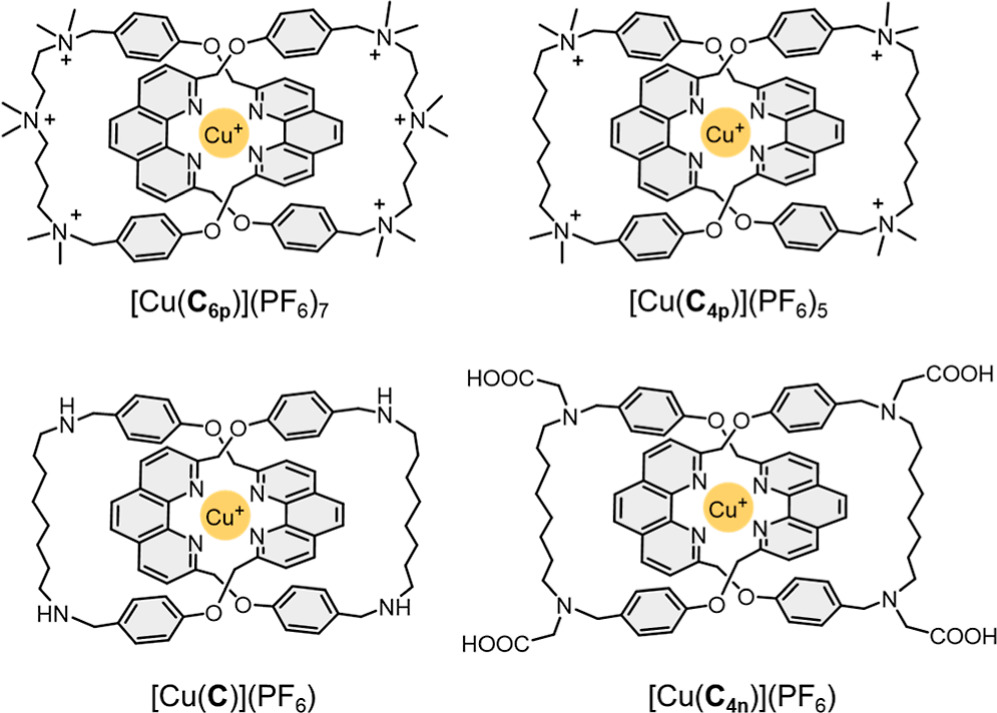
Structures of the Cu(I) catenane electrocatalysts. [Cu(**C**_**6p**_)](PF_6_)_7_ and [Cu(**C**_**4p**_)](PF_6_)_5_ are supported by a [2]catenane ligand that incorporates six and four ammonium units, respectively. [Cu(**C**_**4n**_)](PF_6_) contains four carboxylic acids that exchange with carboxylates under experimental conditions.

A series of Cu(I) catenane complexes, differing by the number of cationic ammonium groups as well as the size of the interlocked macrocycles, were synthesized and studied as electrocatalysts for NO_3_RR (Figures 2 and 6a). Of note, due to the mutual Coulombic repulsion between the cationic ammoniums, the structural integrity of the labile Cu(I) bis(phenanthroline) coordination is maintained only in the presence of the mechanical bond; otherwise, a dynamic mixture of Cu(I) species of various ligand stoichiometry, nuclearity, and number of coordinated solvents will result.^60^ Synthesis of these Cu(I) catenane complexes is detailed in the Supporting Information.^61^

**Figure 3:**
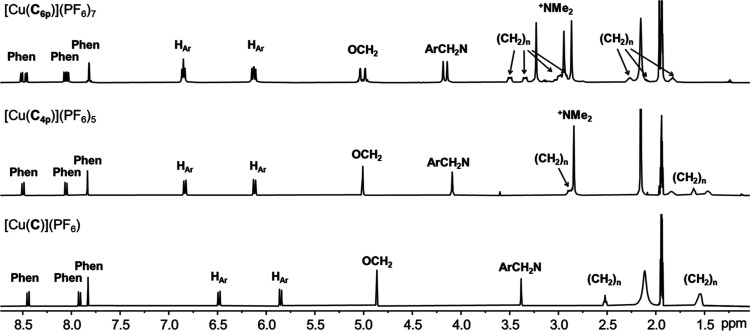
^1^H NMR spectra (500 MHz, CD_3_CN, 298 K) of [Cu(**C**_**6p**_)](PF_6_)_7_, [Cu(**C**_**4p**_)](PF_6_)_5_, and [Cu(**C**)](PF_6_).

Effects of the cationic ammonium groups on the structures of the complexes are studied by ^1^H NMR (Figure 3). Due to the electrostatic repulsion between the ammoniums in [Cu(**C**_**6p**_)](PF_6_)_7_ and [Cu(**C**_**4p**_)](PF_6_)_5_, the interlocked macrocycles in these complexes are likely adopting a more “open” conformation when compared to [Cu(**C**)](PF_6_). More downfield resonances (by 0.28–0.37 ppm) were found for the H_Ar_ protons in [Cu(**C**_**6p**_)](PF_6_)_7_ and [Cu(**C**_**4p**_)](PF_6_)_5_, which is consistent with a looser stacking between the phenyl and phenanthroline units. Of note, the sample of [Cu(**C**_**6p**_)](PF_6_)_7_ is a racemic mixture of opposite mechanical chirality due to the directionality in the macrocycles, which is evidenced by the diastereotopic splitting of the OCH_2_ and ArCH_2_N resonances.

**Figure 4:**
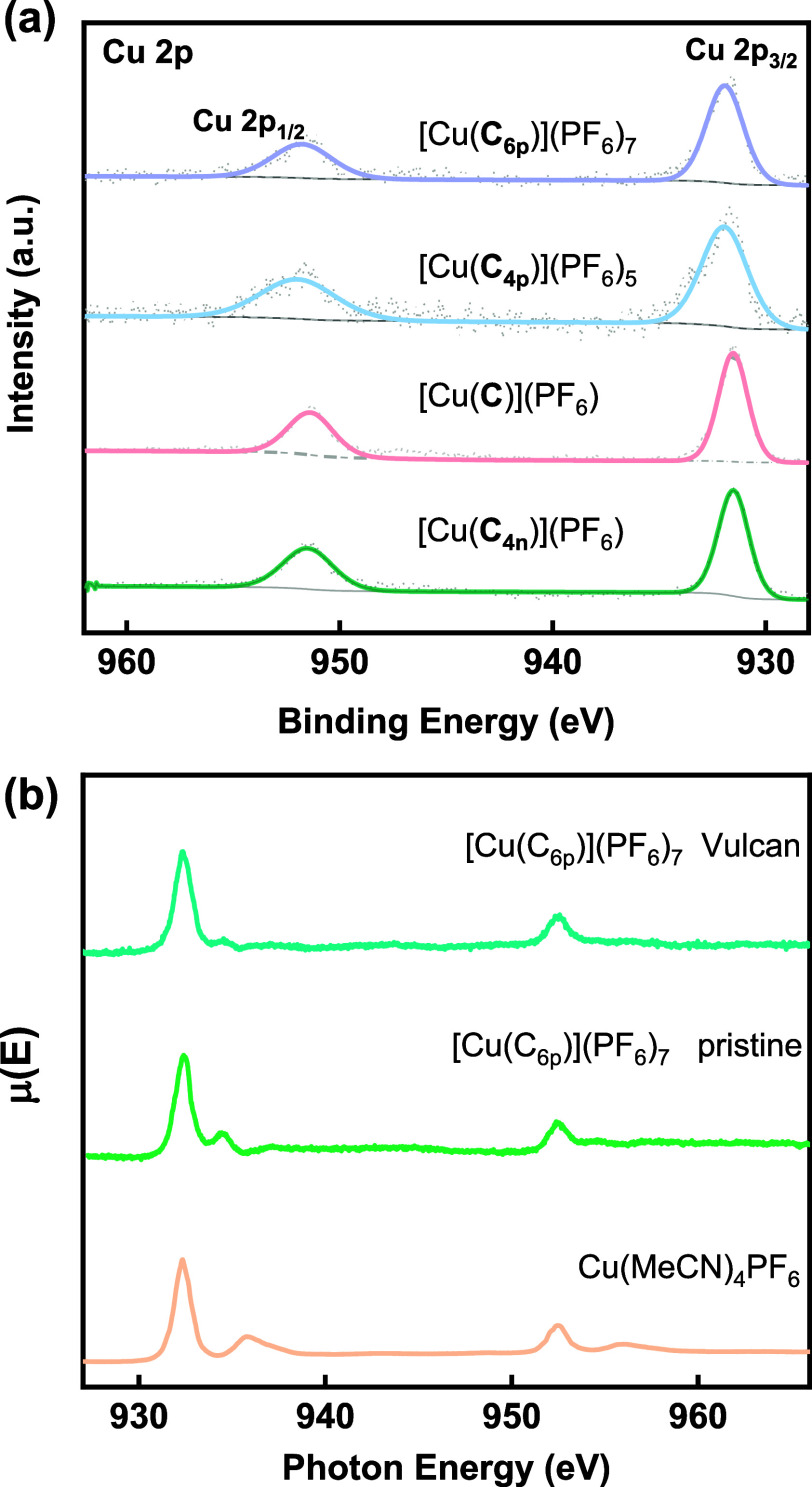
(a) High-resolution X-ray photoelectron spectra (XPS) of Cu 2p in [Cu(**C**_**6p**_)](PF_6_)_7_ (purple), [Cu(**C**_**4p**_)](PF_6_)_5_ (blue), [Cu(**C**)](PF_6_) (pink), and [Cu(**C**_**4n**_)](PF_6_) (green). (b) Cu L3-edge XAS of Cu(MeCN)_4_(PF_6_) reference (orange), [Cu(**C**_**6p**_)](PF_6_)_7_ pristine (green), and [Cu(**C**_**6p**_)](PF_6_)_7_ supported on Vulcan (blue). XAS spectra were collected in TEY mode.

Effects of the cationic ammonium groups on the redox properties of the Cu(I) catenane complexes are explored by X-ray techniques and voltammetry. X-ray photoelectron spectra (XPS) of [Cu(**C**_**6p**_)](PF_6_)_7_, [Cu(**C**_**4p**_)](PF_6_)_5_, [Cu(**C**)](PF_6_), and [Cu(**C**_**4n**_)](PF_6_) showed that Cu is in the 1+ oxidation state (BE = ca. 932 eV) in all these catenanes (Figures 4 and S17). Cu L3-edge X-ray absorption spectroscopy (XAS) conducted in total electron yield (TEY) mode on pristine [Cu(**C**_**6p**_)](PF_6_)_7_ and its carbon-supported version ([Cu(**C**_**6p**_)](PF_6_)_7_/Vulcan) also corroborated a Cu(I) center in pristine [Cu(**C**_**6p**_)](PF_6_)_7_ and [Cu(**C**_**6p**_)](PF_6_)_7_/Vulcan, indicating that the oxidation state of the Cu(I) in the catenanes is not affected upon physical adsorption on mesoporous Vulcan. The electrochemical behavior of [Cu(**C**_**4n**_)](PF_6_), [Cu(**C**)](PF_6_), and [Cu(**C**_**6p**_)](PF_6_)_7_ is further investigated using cyclic voltammetry (CV) in N_2_-saturated, 5× phosphate-buffered saline (PBS) buffer at pH 7. Estimated p*K*_a_ values of the four carboxyl groups in [Cu(**C**_**4n**_)](PF_6_) are 1.6, 2.8, 4.4, and 6.1, suggesting that ca. 90% of the catenane complexes are fully deprotonated with four COO^–^ pendants under the CV condition. The CV curves of [Cu(**C**_**6p**_)](PF_6_)_7_/Vulcan, [Cu(**C**)](PF_6_)/Vulcan, and [Cu(**C**_**4n**_)](PF_6_)/Vulcan showed cathodic peak potentials (*E*_pc_) at 0.62, 0.58, and 0.58 V vs reversible hydrogen electrode (RHE), respectively. The more positive *E*_pc_ of [Cu(**C**_**6p**_)](PF_6_)_7_ suggests that the Cu(II/I) reduction is thermodynamically more favorable with the cationic catenane.^60^ A similar positive shift is also observed when the CV was conducted using unsupported complexes in MeCN (Figure S18).

### Effect of Ammonium Groups on the Electrocatalytic Nitrate Reduction Activity and Product Selectivity of Mechanically Interlocked Cu Complexes

**Figure 5:**
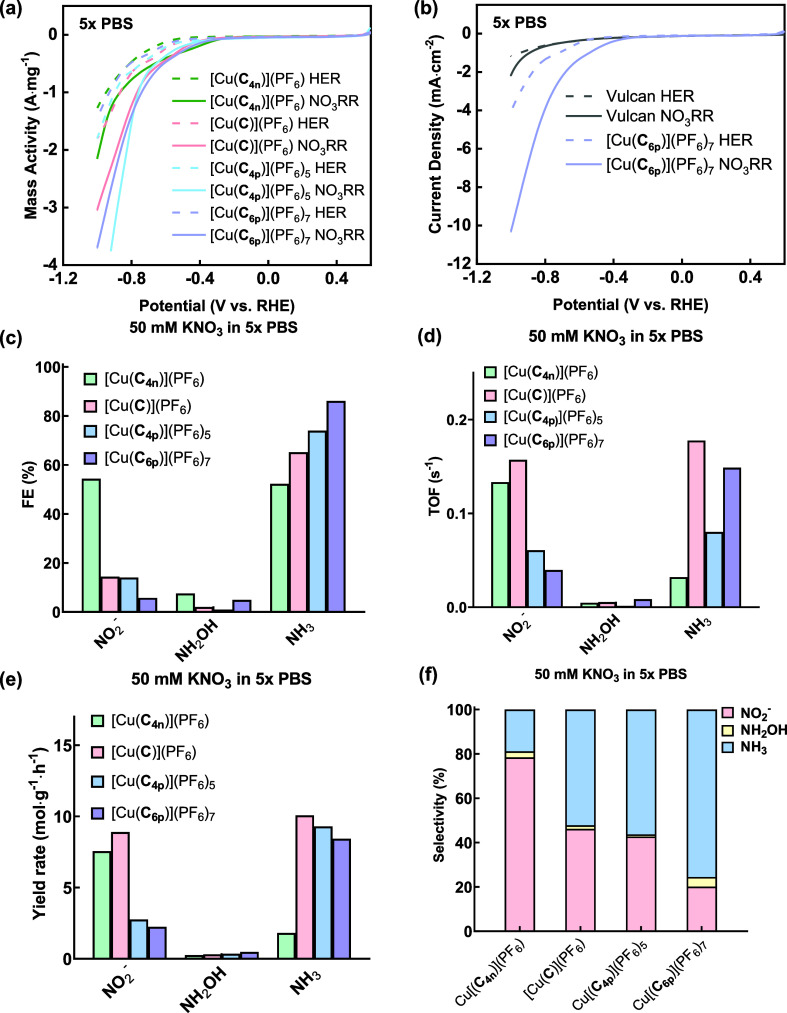
Impact of charged substituents on the electrocatalytic performance of the Cu(I) catenanes. (a) Mass activity of NO_3_RR (solid line) and HER (dashed line) in pH 7 Ar-saturated 5× PBS catalyzed by [Cu(**C**_**4n**_)](PF_6_) (green), [Cu(**C**)](PF_6_) (pink), [Cu(**C**_**4p**_)](PF_6_)_5_ (blue), and [Cu(**C**_**6p**_)](PF_6_)_7_ (purple). (b) Comparison of HER and NO_3_RR performance of the [Cu(**C**_**6p**_)](PF_6_)_7_ catalyst and Vulcan only. (c) Faradaic efficiency (FE), (d) turnover frequency (TOF), and (e) yield rate of NH_3_, NH_2_OH, and NO_2_^–^ obtained by [Cu(**C**_**4n**_)](PF_6_) (green), [Cu(**C**)](PF_6_) (pink), [Cu(**C**_**4p**_)](PF_6_)_5_ (blue), and [Cu(**C**_**6p**_)](PF_6_)_7_ (purple) at −0.8 V vs RHE. (f) Product selectivity of [Cu(**C**_**4n**_)](PF_6_), [Cu(**C**)](PF_6_), [Cu(**C**_**4p**_)](PF_6_)_5_, and [Cu(**C**_**6p**_)](PF_6_)_7_ at −0.8 V vs RHE.

The electrocatalytic nitrate reduction performance and effects of the charged prosthetic groups were evaluated by using [Cu(**C**_**4p**_)](PF_6_)_5_/Vulcan, [Cu(**C**)](PF_6_)/Vulcan, and [Cu(**C**_**4n**_)](PF_6_)/Vulcan as the catalysts by linear sweep voltammetry (LSV). The catalytic activities are normalized on a per mass of Cu basis. In the absence of nitrate, the onset potentials of the hydrogen evolution reaction (HER) for all three Cu(I) catenane complexes are observed at −0.53 V vs RHE (Figure 5a, dashed line). Upon addition of nitrate, all as-prepared Cu(I) complexes displayed a positive shift in the onset potentials to −0.38 V vs RHE, with a concomitant increase in the mass activities, indicating that NO_3_RR likely overtakes HER as the dominant and more facile process when nitrate is present. A significantly higher NO_3_RR activity was found for [Cu(**C**_**4p**_)](PF_6_)_5_ compared with [Cu(**C**)](PF_6_) and [Cu(**C**_**4n**_)](PF_6_)_5_ (Figure 5). In addition, a higher NO_3_^–^ conversion rate (by ∼7-fold) was found for [Cu(**C**_**6p**_)](PF_6_)_7_/Vulcan when compared with the other Cu(I) catalysts (Figure S28a). These results suggest that the positively charged appendages on the catenanes indeed impact the electrocatalytic NO_3_RR activity of the Cu(I) catalysts. Further comparing the NO_3_RR of [Cu(**C**_**6p**_)](PF_6_)_7_ and [Cu(**C**_**4p**_)](PF_6_)_5_, a more positive onset potential (by ∼50 mV) was found in the normalized LSV of [Cu(**C**_**6p**_)](PF_6_)_7_ at the same mass activity of 0.5 A·mg^–1^, corroborating that [Cu(**C**_**6p**_)](PF_6_)_7_, containing the highest number of cationic ammoniums on the catenane, is the most efficient for electrocatalytic NO_3_RR among all of the tested Cu(I) catenanes.

Products generated from the NO_3_RR were next analyzed and compared to further understand the effects of the charged catenanes on the selectivity. After holding the Cu(I) catenane complexes at −0.8 V vs RHE for 30 min (Figure S27), the three main NO_3_RR products, namely, nitrite (NO_2_^–^), hydroxylamine (NH_2_OH), and ammonia (NH_3_), were detected and quantified. For [Cu(**C**_**4n**_)](PF_6_)/Vulcan, similar Faradaic efficiencies (FE) for NO_2_^–^ and NH_3_ were obtained, suggesting a nonselective nitrate reduction. In contrast, [Cu(**C**)](PF_6_)/Vulcan, [Cu(**C**_**4p**_)](PF_6_)_5_/Vulcan, and [Cu(**C**_**6p**_)](PF_6_)_7_/Vulcan exhibited a much higher FE for NH_3_ of ca. 65%, 74%, and 86%, respectively, with a low FE for NO_2_^–^ of ca. 14%, 14%, and 5%, respectively. As such, [Cu(**C**_**6p**_)](PF_6_)_7_ displays the highest selectivity toward NH_3_ (76%) when compared to [Cu(**C**)](PF_6_) (52%), [Cu(**C**_**4p**_)](PF_6_)_5_ (56%), and [Cu(**C**_**4n**_)](PF_6_) (19%) (Figure 5). Only a small amount of NH_2_OH was detected in all of these cases. On the other hand, the turnover frequency (TOF) and yield rate of [Cu(**C**_**6p**_)](PF_6_)_7_ are comparable to those of [Cu(**C**)](PF_6_) and much higher than those of [Cu(**C**_**4n**_)](PF_6_), suggesting that the NO_3_^–^ acceptance is likely assisted by the cationic ammoniums in [Cu(**C**_**4p**_)](PF_6_)_5_ and [Cu(**C**_**6p**_)](PF_6_)_7_ and that the electron and proton transfer during product formation is also promoted. In contrast, the anionic carboxylates in [Cu(**C**_**4n**_)](PF_6_) may lead to an unfavorable microenvironment for NO_3_^–^ absorption, thereby inhibiting the subsequent NO_3_^–^ reduction steps and limiting the overall NH_3_ selectivity. These results highlight the roles of charged prosthetic groups on coordination ligands in control of the activity and selectivity of transition metal catalysts beyond the primary coordination sphere. Similar strategies of exploiting electrostatic interactions in stabilizing reaction intermediates and enhancing catalytic performances of Fe-porphyrins have also been reported.^62,63^

Kinetic isotope effect (KIE) studies were conducted to gain insights into the role of protons during the NO_3_RR. Upon deuteration, minimal negative shifts by only 0.02 V in the onset potentials were observed for the tested Cu(I) catenanes, suggesting a minimal equilibrium isotope effect (Figure S26). For [Cu(**C**_**4n**_)](PF_6_), NO_2_^–^ remained as the dominant product upon deuteration, showing that the carboxylates have no influence on the product selectivity of Cu upon switching to pD 7. In contrast, NO_2_^–^ emerges as the major product for [Cu(**C**)](PF_6_)/Vulcan (72%) and [Cu(**C**_**6p**_)](PF_6_)_7_/Vulcan (75%) in deutero solutions (Figure S26f). A switch in the product selectivity from NQ_3_ (Q = H or D) in proteo solutions to nitrite in deutero solutions indicates that a slow proton/deuteron transfer impedes HER/DER as well as the reduction steps in the NO_3_RR, thereby resulting in a partial conversion of NO_3_^–^ into NO_2_^–^ via a 2e^–^/2H^+^ pathway. Upon switching from deutero to proteo solutions, there is a larger increase in the FE of NQ_3_ for [Cu(**C**_**6p**_)](PF_6_)_7_/Vulcan (∼40%) when compared to [Cu(**C**)](PF_6_)/Vulcan (∼15%). The more drastic increase in the NQ_3_ selectivity exhibited by [Cu(**C**_**6p**_)](PF_6_)_7_/Vulcan corroborates that the cationic ammoniums on the catenanes can facilitate the downstream 8e^–^/9H^+^ steps to give NH_3_ after the initial NO_3_^–^ acceptance by the Cu center.

A higher cathodic current density was observed for [Cu(**C**_**4n**_)](PF_6_)/Vulcan than for [Cu(**C**)](PF_6_)/Vulcan and [Cu(**C**_**6p**_)](PF_6_)_7_/Vulcan at −0.8 V vs RHE in deutero solutions. This trend is opposite to that observed in the proteo cases and can be rationalized also by the selectivity change. Since [Cu(**C**_**4n**_)](PF_6_)/Vulcan prefers the 2e^–^/2H^+^ pathway to generate NO_2_^–^ from NO_3_^–^ in both pH 7 and pD 7, the current density therefore remains similar in both cases. For [Cu(**C**)](PF_6_)/Vulcan and [Cu(**C**_**6p**_)](PF_6_)_7_/Vulcan, since proton transfer is faster than deuteron transfer, there is a switch in the product selectivity from NO_2_^–^ (via 2e^–^/2H^+^ transfer) to NQ_3_ (via 8e^–^/9H^+^ transfer) upon changing from pD 7 to pH 7, and a corresponding increase in the cathodic currents was therefore observed. Moreover, the FE, TOF, and yield rate of NQ_3_ of [Cu(**C**)](PF_6_)/Vulcan and [Cu(**C**_**6p**_)](PF_6_)_7_/Vulcan decreased, while those of nitrite increased in deutero solutions as compared with proteo solutions (Figure S26c–e). Taken together, these observations suggest that the charged appendages in [Cu(**C**_**6p**_)](PF_6_)_7_ and [Cu(**C**_**4n**_)](PF_6_) influence the Cu(I) active site regarding the NO_3_^–^ binding and proton delivery, thereby modulating the NO_3_RR activity and product selectivity.

In addition, the stability and durability of [Cu(**C**_**6p**_)](PF_6_)_7_ (1 mM in MeCN, 100 mM (^*n*^Bu_4_N)(PF_6_)) were evaluated by chronoamperometry experiments (Figure S23). During the course of an 8 h chronoamperometry at −1.2 V vs NHE, a steady current density was observed. Characterizations of [Cu(**C**_**6p**_)](PF_6_)_7_ by CV, UV–vis spectroscopy, ESI–MS, and ^1^H NMR analysis before and after the chronoamperometry experiment indicated that the Cu(I) complex did not decompose and retained its interlocked structure under the operational potential.

### Effect of the Catenane Ring Size on Electrocatalytic Nitrate Reduction Activity and Product Selectivity

**Figure 6:**
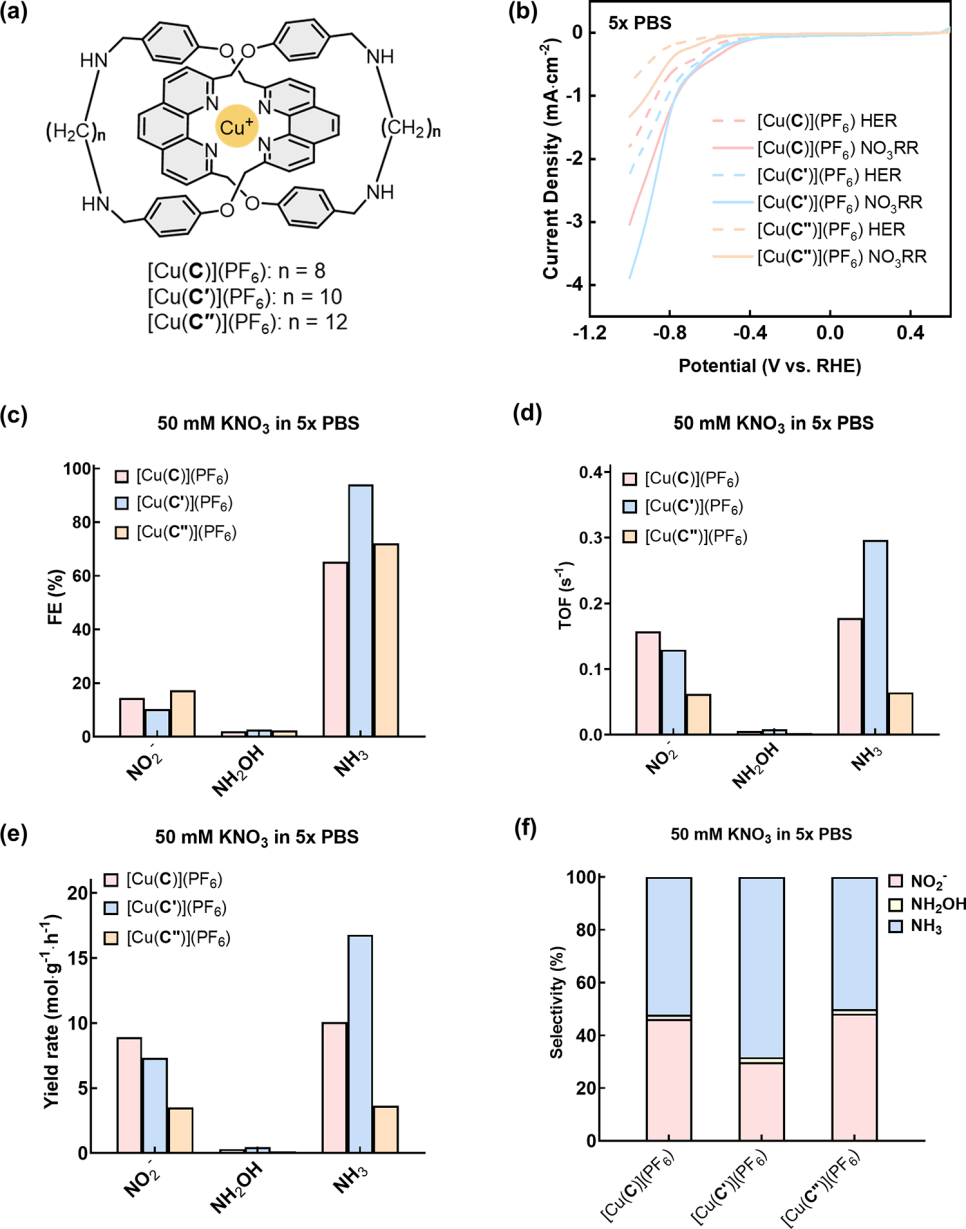
Impact of mechanical bond tightness on the electrocatalytic performance of Cu catenanes. (a) Structures of Cu(I) catenane complexes with different sizes of the interlocked macrocycles. (b) Mass activity with NO_3_RR (solid line) and HER (dashed line) in pH 7 Ar-saturated 5× PBS catalyzed by [Cu(**C**)](PF_6_) (pink), [Cu(**C′**)](PF_6_) (blue), and [Cu(**C″**)](PF_6_) (orange). (c) Faradaic efficiency (FE), (d) turnover frequency (TOF), and (e) yield rates of NH_3_, NH_2_OH, and NO_2_^–^ obtained by [Cu(**C**)](PF_6_) (pink), [Cu(**C′**)](PF_6_) (blue), and [Cu(**C″**)](PF_6_) (orange) at −0.8 V vs RHE. (f) Product selectivity of [Cu(**C**)](PF_6_), [Cu(**C′**)](PF_6_), and [Cu(**C″**)](PF_6_) at −0.8 V vs RHE.

Because [Cu(**C**)](PF_6_)/Vulcan exhibited a high TOF and yield rate for nitrate-to-ammonia reduction, Cu(I) catenanes analogous to [Cu(**C**)](PF_6_) were further explored to study the relationship between ligand interlocking and the NO_3_RR performance. In particular, kinetics and thermodynamics of the tetrahedral-to-planar geometry change involving Cu(I)-to-Cu(II) transition during PCET are likely to be influenced by the ability of the catenane to undergo co-conformational changes. The NO_3_RR performance can therefore be controlled by varying the length of the flexible aliphatic linkers that affect the co-conformational flexibility of the Cu(I) catenanes. Specifically, Vulcan-supported [Cu(**C**)](PF_6_), [Cu(**C′**)](PF_6_), and [Cu(**C″**)](PF_6_), featuring, respectively, C_8_, C_10_, and C_12_ linkers in the catenane backbone, were investigated as electrocatalysts for NO_3_RR, and their FE, TOF, yield rate, and product selectivity are shown in Figure 6. Although there is a similar onset potential (−0.38 V vs RHE) for [Cu(**C**)](PF_6_) and [Cu(**C′**)](PF_6_), the cathodic current density of [Cu(**C′**)](PF_6_) is higher than that of [Cu(**C**)](PF_6_). On the other hand, [Cu(**C″**)](PF_6_) is found to be less active in the NO_3_RR with an additional 200 mV overpotential when compared with [Cu(**C**)](PF_6_) and [Cu(**C′**)](PF_6_). While NH_3_ remains as the major product for all three Cu(I) catenane catalysts, [Cu(**C′**)](PF_6_) exhibited the highest FE (95%), TOF (0.30 s^–1^), yield rate (17 mol g^–1^ h^–1^), and hence the best NH_3_ selectivity (70%). The NO_3_^–^ conversion rate of [Cu(**C′**)](PF_6_) is also the highest among the three Cu(I) catenanes (Figure S28b). The trend in the NO_3_RR activity and product selectivity could be rationalized by the energetics of the [2]catenanes in undergoing (co)conformational changes upon accepting NO_3_^–^, as well as mediating downstream reduction and intermediate conversion, and a similar dependence of the activity and selectivity on the catenane ring size is also observed previously.^56,57,60^

## Conclusions

A series of molecular Cu(I) complexes supported by catenane ligands tailored for efficient electrocatalytic NO_3_RR has been developed. By mechanical interlocking, cationic ammoniums were successfully introduced to the catenane skeleton to enhance the activity and selectivity of ammonia formation while maintaining the structural integrity of the catalysts. Electrochemical studies showed that [Cu(**C**_**6p**_)](PF_6_)_7_, featuring a hexacationic catenane ligand, can selectively generate ammonia with a Faradaic efficiency reaching 86%, while the anionic control [i.e., [Cu(**C**_**4n**_)](PF_6_)] exhibited suppressed activity and selectivity, highlighting the benefits of introducing the positively charged groups in the NO_3_RR. In addition, the NO_3_RR performance of the Cu(I) catenanes was also found to be dependent on the size of the interlocked macrocycles. Taken together, these results demonstrated that the NO_3_RR performance of the Cu catalysts can be enhanced via both covalent modification (chemical) and mechanical interlocking (physical) of the supporting ligands, providing new directions of catalyst design for further improvement of the NO_3_RR performance. These results are valuable not only because of the rational design of Cu(I) complexes for efficient electrocatalytic reactions involving intricate proton-coupled electron transfer steps but also because of the potential of mechanically interlocked ligands as a platform for transition metal catalyst customization with molecular features that are not easily achievable by noninterlocked analogues and conventional catalyst designs.^56,57,60^

## Methods

### General Methods

Chemicals for molecular synthesis were obtained from commercial sources and used as received unless otherwise stated. Sodium hydroxide (NaOH, analytical grade, Merck Millipore), Nafion perfluorinated resin solution (5 wt % in lower aliphatic alcohols and water, containing 15–20% water, Sigma-Aldrich), sodium nitrite (NaNO_2_, Acros Organics), ammonium chloride (NH_4_Cl, A.R. Dieckmann), phenol (C_6_H_5_OH, Sigma-Aldrich), sodium citrate anhydrous (Na_3_C_6_H_5_O_7_, J&K Scientific), sodium hypochlorite solution (NaClO solution, 11–14% available chlorine, Alfa Aesar), and sodium nitroprusside [Na_2_Fe(CN)_5_NO·2H_2_O, A.R. Beijing Huagongchang]. Electrochemical studies at pH 7 were performed in PBS buffer containing sodium chloride (NaCl, Dieckmann), potassium chloride (KCl, J&K Scientific), sodium hydrogen phosphate (Na_2_HPO_4_, Dieckmann), and potassium dihydrogen phosphate (KH_2_PO_4_, Dieckmann). NO_3_RR experiments were conducted in the above PBS buffer with 50 mM potassium nitrate (KNO_3_, Acros Organics). All buffer solutions were prepared using Milli-Q water (>18 MΩ cm) and were sparged with Ar (99.995% high purity grade, Linde HKO) for 30 min before each experiment following published methods.^34,57,64^

### General Inks Preparation Method

Vulcan XC-72 carbon black (Cabot) was pretreated and soaked in 0.1 M HCl (Duksan, 37% GR) for 24 h. The acid-treated carbon black was filtered and washed using Milli-Q ultrapure water and then dried in a vacuum oven at 80 °C overnight. [Cu(**C**_**4n**_)](PF_6_), [Cu(**C**_**4p**_)](PF_6_)_5_, [Cu(**C**_**6p**_)](PF_6_)_7_, [Cu(**C**)](PF_6_), [Cu(**C′**)](PF_6_), and [Cu(**C″**)](PF_6_) were fully dissolved in acetonitrile (RCI Labscan, AR) and mixed with the pretreated Vulcan XC-72 to form their respective carbon mixtures (30% w/w). The mixtures were sonicated for 5 min. After sonication, the mixtures were dried under vacuum at 37 °C overnight. Finely ground [Cu(**C**_**4n**_)](PF_6_), [Cu(**C**_**4p**_)](PF_6_)_5_, [Cu(**C**_**6p**_)](PF_6_)_7_, [Cu(**C**)](PF_6_), [Cu(**C′**)](PF_6_), and [Cu(**C″**)](PF_6_) catalysts on Vulcan XC-72 (4 mg) were suspended and sonicated in 1 mL of ethanol (Scharlab, Abs) for 15 min, respectively. Nafion perfluorinated resin solution (4 μL, 5 wt % in lower aliphatic alcohols with 15–20% water, Sigma-Aldrich) was added into the well-dispersed catalyst/Vulcan in ethanol. The resulting mixture was then continuously sonicated for 10 min to form inks. Eight μL of the ink was drop-casted onto a glassy carbon electrode (*A* = 0.07065 cm^2^, Gaoss Union), which was polished with a 3–0.5 μm alumina suspension polishing kit (Allied Tech) and further dried under a stream of N_2_.^57,65,66^

### Electrochemical Activity Measurements

Electrochemical studies were carried out using a CH Instruments 760E electrochemical workstation at room temperature following the published procedures. Experiments were performed in a three-compartment cell with an aqueous Ag/AgCl (3 M KCl, CHI) reference electrode separated from the working electrode by a Luggin capillary as described previously.^67^ Electrochemical potentials were reported relative to the RHE by using a published protocol. A Pt-wire counter electrode was separated from the working electrode with a glass frit. Chronoamperometric measurements were conducted at −0.8 V vs RHE in 5× PBS (685 mM NaCl, 13.5 mM KCl, 50 mM Na_2_HPO_4_ and 9 mM KH_2_PO_4_) buffer solution containing 50 mM KNO_3_ for 0.5 h. The volume of the electrolyte in each chamber was 4 mL. Before electrochemical tests, the electrolyte solution was sparged with Ar for 30 min to remove dissolved O_2_. LSV was conducted at a scan rate of 10 mV s^–1^. Electrolysis was performed at a given potential for a designated time period.^34^

### Material Characterization

XPS was conducted using a Thermo Scientific ESCALAB QXi^+^ XPS microprobe, and the spectra were calibrated with a binding energy of 284.8 eV for C 1s. The data were analyzed following a published protocol.^65,67^ Ex-situ Cu L3-edge X-ray absorption spectroscopy (XAS) measurements were conducted in TEY mode at the TLS beamline 20A end station at the National Synchrotron Radiation Research Center (NSRRC).^68^

### Product Analysis

Nitrate and nitrite concentrations were quantified by using an ion chromatograph (IC) system (Thermo Scientific ICS-1100) with an electrochemical detector (ECD). 1000 ppm of NO_3_^–^ and NO_2_^–^ stock solutions were prepared in Milli-Q ultrapure water, diluted to known concentrations, and injected into the IC system to obtain a series of peak areas. Standard curves were prepared by correlating the NO_3_^–^ and NO_2_^–^ concentrations and the peak areas.^34^

All UV–visible spectra were collected using an Implen Nanophotometer NP80 UV–vis spectrophotometer with UVette (Eppendorf) cuvettes. Ammonia was quantified by an indophenol blue method.^69^ Phenol (1.058 g) was dissolved in absolute ethanol (10 mL) to prepare a fresh phenol solution, which can be used for a week. Sodium nitroprusside (0.05 g) was dissolved in Milli-Q water (10 mL) to prepare an Fe catalyst solution. 5% NaClO solution was diluted from commercially available 11–14% NaClO using Milli-Q water. The alkaline citrate solution was prepared by dissolving NaOH (2.5 g) and trisodium citrate (50 g) in a Milli-Q water (250 mL). The alkaline oxidizing solution was prepared by mixing the alkaline citrate solution and 5% NaClO solution with a V/V ratio of 4:1. Ammonia standard solutions were prepared by diluting NH_4_Cl stock solution (1000 ppm) into designated concentrations. Standard solution (0.5 mL) was diluted 5-fold using Milli-Q water. Phenol solution (0.1 mL), Fe catalyst solution (0.1 mL), and alkaline oxidizing solution (0.425 mL) were sequentially added to the diluted standard solution. After it was mixed thoroughly, the final solution was placed in the dark at room temperature for 4 h. The NH_4_^+^ standard curve was constructed by correlating standard solution concentrations with absorbances at 630 nm.^34,38,70^ The same procedure was used to quantify the amount of NH_4_^+^ in the electrolysis experiment.

Hydroxylamine was quantified by a revised method with K_3_Fe(CN)_6_ under strong alkaline conditions. 0.2 mL of sample or NH_2_OH standard, 0.1 mL of K_3_Fe(CN)_6_ in 100 mM KCl, and 0.3 mL of 25% KOH were mixed thoroughly following the writing sequence and reacted for 7 min. A blank control was conducted by replacing the sample with electrolyte only. The NH_2_OH standard curve was constructed by correlating standard solution concentrations with absorbances at 425 nm.^70^

### Product Analysis Calculations

Faradaic efficiency (%)
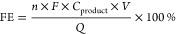
1where *C*_product_ is the concentration of each product in mol L^–1^, *V* is the volume of electrolyte solution (4 × 10^–3^ L), *n* is the number of electrons required to obtain a certain product, *F* is the Faraday constant (96,485 C mol^–1^), and *Q* is the total charge (*C*) passed during electrolysis.

Turnover frequency (s^–1^)
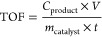
2where *C*_product_ is the concentration of each product in mol L^–1^, *V* is the volume of electrolyte solution (4 × 10^–3^ L), *F* is the Faraday constant (96485 C mol^–1^), *m* is the number of moles of catalyst, and *t* is the electrolysis time in s.

Yield rate (mol g^–1^ h^–1^)
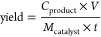
3where *C*_product_ is the concentration of each product in mol L^–1^, *V* is the volume of electrolyte solution (4 × 10^–3^ L), *M*_catalyst_ is the mass of the Cu catalyst, and *t* is the electrolysis time in h.

Product selectivity (%)
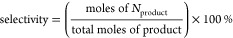
4*N*_product_ refers to the nitrogenous product generated.

NO_3_^–^ conversion rate (%)
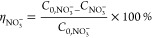
5

### Methods for KIE Studies

All proteo and deutero aqueous solutions were prepared freshly each day using Milli-Q water (>18 MΩ cm) and D_2_O, respectively. For experiments in pH 7 and pD 7, both proteo and deutero PBS solutions containing sodium chloride (NaCl, Dieckmann), potassium chloride (KCl, J&K Scientific), sodium hydrogen phosphate (Na_2_HPO_4_, Dieckmann), and potassium dihydrogen phosphate (KH_2_PO_4_, Dieckmann) were used. NO_3_RR experiments were conducted in the PBS buffer described above with 50 mM potassium nitrate (KNO_3_, Acros Organics). The pH and pD values of buffer solutions were measured using a Jenway 3510 Standard Digital pH Meter calibrated with three standard buffer solutions.^67,71−73^ pH readings were converted to pD using eq 6, in which pH_a_ is the apparent reading from the pH meter
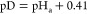
6

## Supporting Information Available

Experimental data, as well as the characterization data for all of the compounds prepared in the course of these studies, experimental details, synthetic procedures, NMR and MS characterization data, control experiments, KIE studies, XPS spectra, ICP–MS results, cyclic voltammograms, linear sweep voltammograms, computational methods and results, supplementary tables, additional notes, and supporting figures (PDF)
