# Supplementary material for: Mechanical and Covalent Tailoring of Copper Catenanes for Selective Aqueous Nitrate-to-Ammonia Electrocatalysis
Source: J Am Chem Soc. 2025 Apr 22;147(17):14316–25. doi: 10.1021/jacs.4c18547 (PMC12046556; doi:10.1021/jacs.4c18547)
Supplement: Supplementary file 1 — ja4c18547_si_001.pdf [file ja4c18547_si_001.pdf]

Supporting Information

Mechanical and Covalent Tailoring of Copper Catenanes for Selective Aqueous Nitrate-to-Ammonia Electrocatalysis

Yulin Deng[1,‡], Xiaoyong Mo[1,‡], Samuel Kin-Man Lai[1], Shu-Chih Haw[2], Ho Yu Au-Yeung[1,3,*], Edmund C. M. Tse[1,*]

[1] HKU-CAS Joint Laboratory on New Materials & Department of Chemistry, The University of Hong Kong, Pokfulam Road, Hong Kong SAR, P. R. China

[2] National Synchrotron Radiation Research Center, 101 Hsin-Ann Road, Hsinchu 30076, Taiwan

[3] State Key Laboratory of Synthetic Chemistry, The University of Hong Kong, Pokfulam Road, Hong Kong SAR, P. R. China

[‡] Equal contribution

[*] Corresponding authors: HYAY: hoyuay@hku.hk; ECMT: ecmtse@hku.hk

Table of Contents

1. Molecular Synthesis and Characterization
2. Physical Characterization and Electrochemical Results
3. Product Analysis
4. Kinetic Isotope Effect Studies
5. Computational Investigation
6. Supplementary References

1. Molecular Synthesis and Characterization

General Methods

All reagents were purchased from commercial suppliers (J & K, Sigma-Aldrich, TCI, Energy, Macklin, Dieckmann, and Cambridge Isotope Laboratories Inc.) and used without further purification unless otherwise noted. All solvents were of analytical grade (ACI Labscan and DUKSAN Pure Chemicals). Building block **Phen-CHO**, Cu(I) complex [Cu(**L**)$_2$](PF$_6$), [2]catenanes [Cu(**C**)](PF$_6$), [Cu(**C′**)](PF$_6$), [Cu(**C″**)](PF$_6$), and [Cu(**C$_{4p}$**)](PF$_6$)$_5$ were synthesized according to literature procedures.[1-3] Thin layer chromatography (TLC) was performed on silica gel 60 F254 (Merck, Germany, Aluminium sheet) and column chromatography was carried out using silica gel 60F (Silicycle, Canada). UPLC-ESI-MS were carried out using a Waters-Acquity UPLC H-Class system coupled with a QDa MS detector. HRMS spectra were obtained from a Bruker Impact II Ultra-High Resolution QTOF mass spectrometer. UV-Vis spectra were recorded using a Varian Cary 50 UV-Vis Spectrophotometer. NMR spectra were recorded on Bruker DPX spectrometers with working frequencies of 400 MHz or 500 MHz for ^{1}H, and 101 MHz or 126 MHz for ^{13}C, respectively. Chemical shifts are reported in ppm and referenced to solvent residues (for ^{1}H: CDCl$_3$: δ = 7.26 ppm, CD$_3$CN: δ = 1.94 ppm, CD$_3$OD: δ = 3.31 ppm; for ^{13}C: CDCl$_3$: δ = 77.16 ppm, CD$_3$CN: δ = 1.32 ppm, CD$_3$OD: δ = 49.00 ppm).

1.1 Synthesis

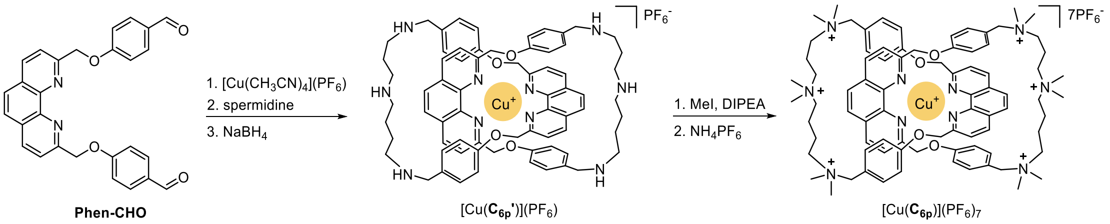

*Synthesis of [Cu(**C$_{6p}$**′)](PF$_6$)*

Under an argon atmosphere, a mixture of **Phen-CHO** (0.54 g, 1.2 mmol) and [Cu(CH$_3$CN)$_4$](PF$_6$) (0.22 g, 0.6 mmol) in 300 mL of 5:3:2 CHCl$_3$/CH$_3$CN/CH$_3$OH (150 mL/90 mL/60 mL) was stirred for 30 minutes at room temperature, after which spermidine (0.26 g, 1.8 mmol) and piperidine (0.5 mL) were added and the reaction mixture was heated at 60 °C for 10 hours. The reaction mixture was cooled to 0 °C in an ice bath, NaBH$_4$ (0.14 g, 3.6 mmol) was added in portions and stirred in the ice bath for 1 hour. The mixture was warmed to room temperature and solvents were removed by a rotary evaporator. The dark red residue was re-dissolved in 50 mL CH$_2$Cl$_2$, washed with water (2 × 100 mL) and brine (100 mL), dried over anhydrous Na$_2$SO$_4$ and filtered. Solvents from the

filtrate were removed by a rotary evaporator to afford an orange solid, which was washed by ethyl acetate (3 × 20 mL), re-dissolved in 30 mL CH_3CN, filtered and concentrated using a rotary evaporator to ~5 mL. Et_2O vapour was diffused to the concentrated solution in CH_3CN to give orange crystalline solids. The solvent was decanted and the product was rinsed by Et_2O (2 mL), followed by drying under vacuum to afford [Cu(**C$_{6p}$′**)](PF_6). Yield = 0.53 g, 66%. ^{1}H NMR (400 MHz, CD_3CN, 298 K) δ 8.51 (d, *J* = 5.8 Hz, 2H), 8.49 (d, *J* = 5.8 Hz, 2H), 7.94–7.90 (m, 8H), 6.48 (dd, *J* = 8.6, 3.9 Hz, 8H), 5.92 (d, *J* = 8.6 Hz, 4H), 5.79 (d, *J* = 8.6 Hz, 4H), 4.93 (s, 4H), 4.80 (s, 4H), 3.42 (s, 4H), 3.36 (s, 4H), 2.81 (t, *J* = 6.5 Hz, 4H), 2.74 (t, *J* = 7.2 Hz, 4H), 2.63 (t, *J* = 6.8 Hz, 4H), 2.46 (t, *J* = 6.4 Hz, 4H), 1.74 (t, *J* = 6.7 Hz, 4H), 1.69–1.63 (m, 4H), 1.60–1.55 (m, 4H). ^{13}C NMR (126 MHz, CD_3CN, 298 K) δ 157.4, 157.1, 155.3, 155.1, 144.1, 144.1, 138.7, 138.7, 134.2, 134.2, 129.8, 129.8, 129.1, 129.0, 127.7, 127.7, 126.5, 126.3, 113.6, 113.3, 72.2, 71.9, 53.8, 53.5, 51.2, 49.8, 49.6, 49.3, 31.7, 29.0, 28.6. HRMS (ESI+): *m*/*z* $[M\text{-}PF_6]^+$ calcd for $C_{70}H_{78}N_{10}O_4CuPF_6$: 1185.5498; found: 1185.5479. UV-Vis absorption (298 K, MeCN): λ_{max} = 277 nm (ε = 46000 M^{-1} cm^{-1}), 461 nm (ε = 5300 M^{-1} cm^{-1}).

***Synthesis of** [Cu(**C$_{6p}$**)](PF_6)$_7$*

Under an argon atmosphere, a mixture of [Cu(**C$_{6p}$′**)](PF_6) (0.32 g, 0.24 mmol), MeI (0.36 mL, 5.8 mmol), *N*,*N*-diisopropylethylamine (0.63 mL, 3.6 mmol) in 50 mL of MeCN was heated at 75 °C for 10 hours. The reaction mixture was filtered, the solid was washed with MeCN (3 × 5 mL) and re-dissolved in de-ionized water (30 mL). NH_4PF_6 (2 g, 12.3 mmol) was added to the aqueous solution, stirred at room temperature for 10 minutes. The obtained precipitation was collected by centrifugation at 4000 rpm for 10 minutes, washed with de-ionized water (3 × 10 mL) and was dried under vacuum. [Cu(**C$_{6p}$**)](PF_6)$_7$ was obtained as an orange solid. Yield = 0.44 g, 78%. ^{1}H NMR (500 MHz, CD_3CN, 298 K) δ 8.51 (d, *J* = 8.1 Hz, 2H), 8.46 (d, *J* = 8.1 Hz, 2H), 8.07 (d, *J* = 8.2 Hz, 2H), 8.04 (d, *J* = 8.1 Hz, 2H), 7.82 (d, *J* = 1.8 Hz, 4H), 6.87–6.82 (m, 8H), 6.13 (dd, *J* = 11.6, 8.7 Hz, 8H), 5.06–5.01 (m, 4H), 5.01–4.96 (m, 4H), 4.18 (s, 4H), 4.14 (s, 4H), 3.50 (t, *J* = 8.5 Hz, 4H), 3.34 (t, *J* = 8.5 Hz, 4H), 3.23 (s, 12H), 3.01–2.96 (m, 4H), 2.94 (s, 12H), 2.94–2.91 (m, 4H), 2.86 (s, 12H), 2.27 (br, 4H), 1.91 (br, 4H), 1.83 (br, 4H). ^{13}C NMR (101 MHz, CD_3CN, 298 K) δ 160.2, 160.1, 155.2, 155.1, 144.2, 144.1, 138.6, 138.4, 135.2, 135.1, 129.5, 129.4, 127.4, 127.2, 127.1, 120.0, 119.9, 114.8, 114.6, 72.1, 71.9, 71.5, 71.1, 64.1, 62.1, 59.3, 59.2, 53.3, 50.4, 50.2, 21.3, 19.9, 17.8. HRMS (ESI+): *m*/*z* $[M\text{-}2PF_6]^{2+}$ calcd for $C_{82}H_{108}N_{10}O_4CuP_7F_{42}$: 1042.3025; found: 1042.2993. Purity by ^{1}H NMR

(comparing the peak integrations with the internal standard 1,3,5-trimethoxybenzene): 99%. Purity by UPLC-MS (by peak integration): 99%. UV-Vis absorption (298 K, MeCN): λ_{max} = 277 nm (ε = 46000 M^{-1} cm^{-1}), 466 nm (ε = 5700 M^{-1} cm^{-1}).

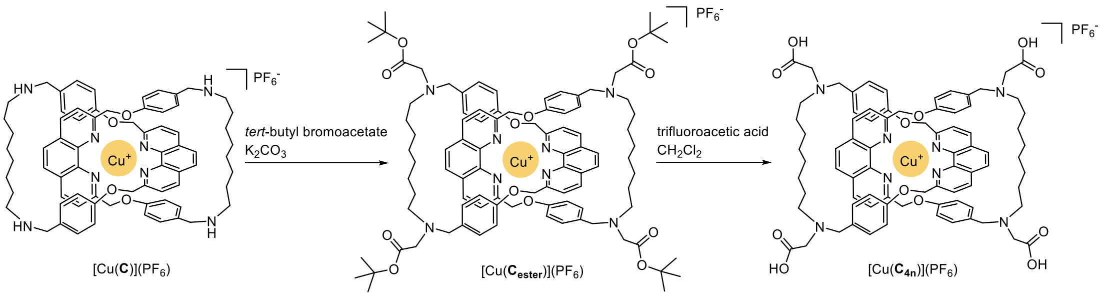

Synthesis of *[Cu(**C**ester)](PF_6)*

Under an argon atmosphere, a mixture of [2]catenane [Cu(**C**)](PF_6) (6.7 mg, 5 μmol), K_2CO_3 (6.9 mg, 0.05 mmol), *tert*-butyl bromoacetate (7.8 mg, 0.04 mmol) in MeCN (2 mL) was heated at 75 °C for 10 hours. The reaction mixture was diluted with CH_2Cl_2 (5 mL), washed with water (5 mL × 2) and brine (5 mL), dried over Na_2SO_4 and filtered. Solvents from the filtrates were removed by a rotary evaporator, and the residue was re-dissolved in CH_2Cl_2 (1 mL). Trifluoroacetic acid (30 μL) was added and the solution was stirred at room temperature for 1 hour. Solvents were evaporated and the residue was re-dissolved in CH_2Cl_2 (0.5 mL), into which Et_2O vapour was slowly diffused to afford orange precipitation. The obtained precipitation was collected by centrifugation at 4000 rpm for 3 minutes and was dried under vacuum. Product was obtained as an orange solid. Yield = 10.0 mg, 92%. ^{1}H NMR (400 MHz, $CDCl_3$, 298 K) δ 8.60 (d, *J* = 8.0 Hz, 4H), 7.99 (s, 4H), 7.93 (d, *J* = 8.1 Hz, 4H), 6.88 (d, *J* = 8.1 Hz, 8H), 5.96 (d, *J* = 7.9 Hz, 8H), 4.84 (s, 8H), 4.15 (s, 8H), 3.71 (s, 8H), 3.04 (br, 8H), 1.70 (br, 8H), 1.47 (s, 36H), 1.40–1.22 (m, 16H). ^{13}C{^{1}H} NMR (101 MHz, $CDCl_3$, 298 K) δ 164.8, 158.4, 154.3, 143.2, 138.5, 132.5, 129.1, 127.2, 125.8, 122.5, 114.0, 84.7, 71.3, 57.6, 51.7, 28.3, 28.0, 27.9, 26.1, 24.8. HRMS (ESI+): *m/z* $[M-PF_6]^+$ calcd for $C_{96}H_{120}N_8O_{12}CuPF_6$: 1640.8348; found: 1640.8331.

Synthesis of *[Cu(**C**4n)](PF_6)*

To a solution of [Cu(***C**ester*)](PF_6) (17.9 mg, 0.01 mmol) in CH_2Cl_2 (1 mL), trifluoroacetic acid (1 mL) was added at 0 °C, and the reaction mixture was stirred at room temperature for 10 hours. Solvents were evaporated by a rotary evaporator. Re-dissolved in 1 mL MeCN, to which $[Cu(MeCN)_4]PF_6$ (3.7 mg,

0.01 mmol) was added and the resulting mixture was stirred at room temperature for 1 hour under argon. The reaction mixture was filtered and Et_2O (5 mL) was added to the filtrate, the precipitates formed were collected by centrifugation at 4000 rpm for 3 minutes and dried under vacuum. The collected solid was redissolved in 0.5 mL MeOH, Et_2O vapour was diffused to the MeOH solution to give orange crystalline solids. The solvents were decanted and the product was rinsed by Et_2O (2 mL), followed by drying under vacuum to afford pure sample of [Cu(**C_{4n}**)](PF_6). Yield = 13.1 mg, 84%. ^{1}H NMR (500 MHz, MeOD, 298 K) δ 8.68 (d, J = 8.2 Hz, 4H), 8.16 (d, J = 8.2 Hz, 4H), 8.04 (s, 4H), 6.95 (d, J = 8.5 Hz, 8H), 6.15 (d, J = 8.7 Hz, 8H), 5.05 (s, 8H), 4.18 (s, 8H), 3.74 (s, 8H), 2.94 (t, J = 8.2 Hz, 8H), 1.83 (br, 8H), 1.56 (br, 8H), 1.49 (br, 8H). ^{13}C NMR (126 MHz, MeOD, 298 K) δ 160.1, 156.1, 144.6, 139.2, 133.7, 130.3, 128.1, 127.3, 122.7, 115.2, 72.3, 58.7, 54.1, 29.3, 27.3, 25.0. HRMS (ESI+): m/z $[M-PF_6+H]^{2+}$ calcd for $C_{80}H_{88}N_8O_{12}CuPF_6$: 708.2942; found: 708.2935. Purity by ^{1}H NMR (comparing the peak integrations with the internal standard 1,3,5-trimethoxybenzene): 99%. Purity by UPLC-MS (by peak integration): 98%. UV-Vis absorption (298 K, MeCN): λ_{max} = 277 nm (ε = 41000 M^{-1} cm^{-1}), 461 nm (ε = 5100 M^{-1} cm^{-1}).

1.2 NMR Spectroscopy

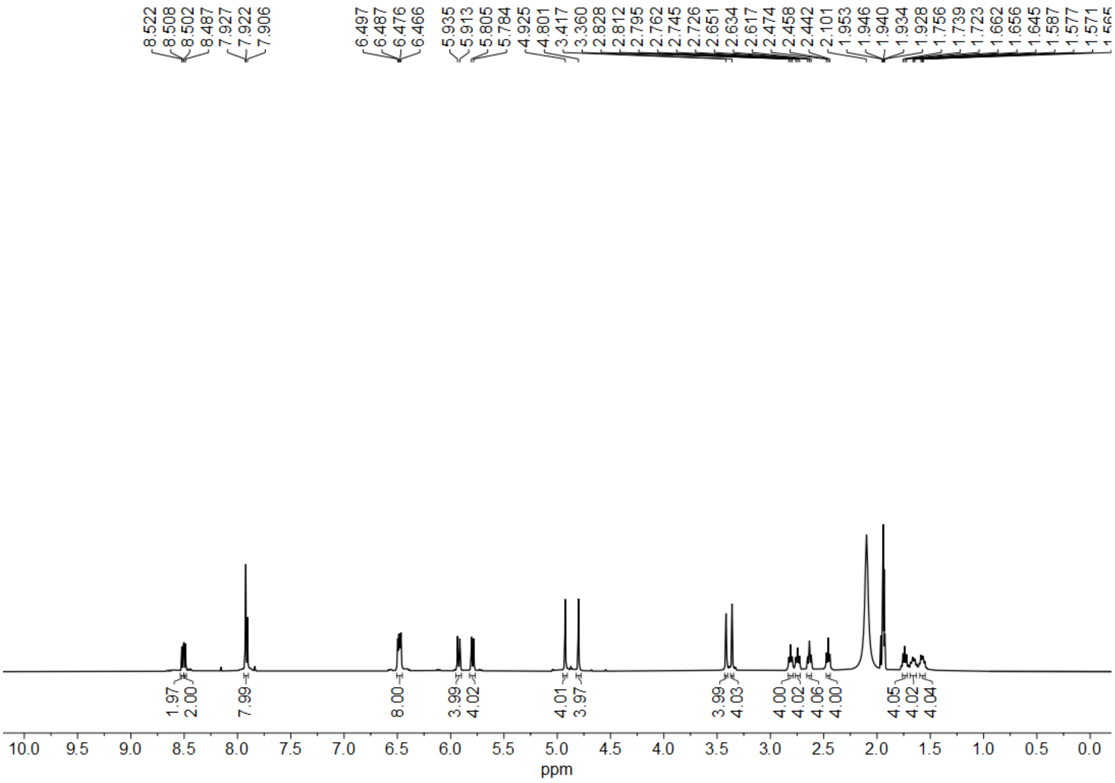

Figure S1. ^{1}H NMR (400 MHz, CD_3CN, 298 K) of [Cu(**C$_{6p}$′**)](PF$_6$).

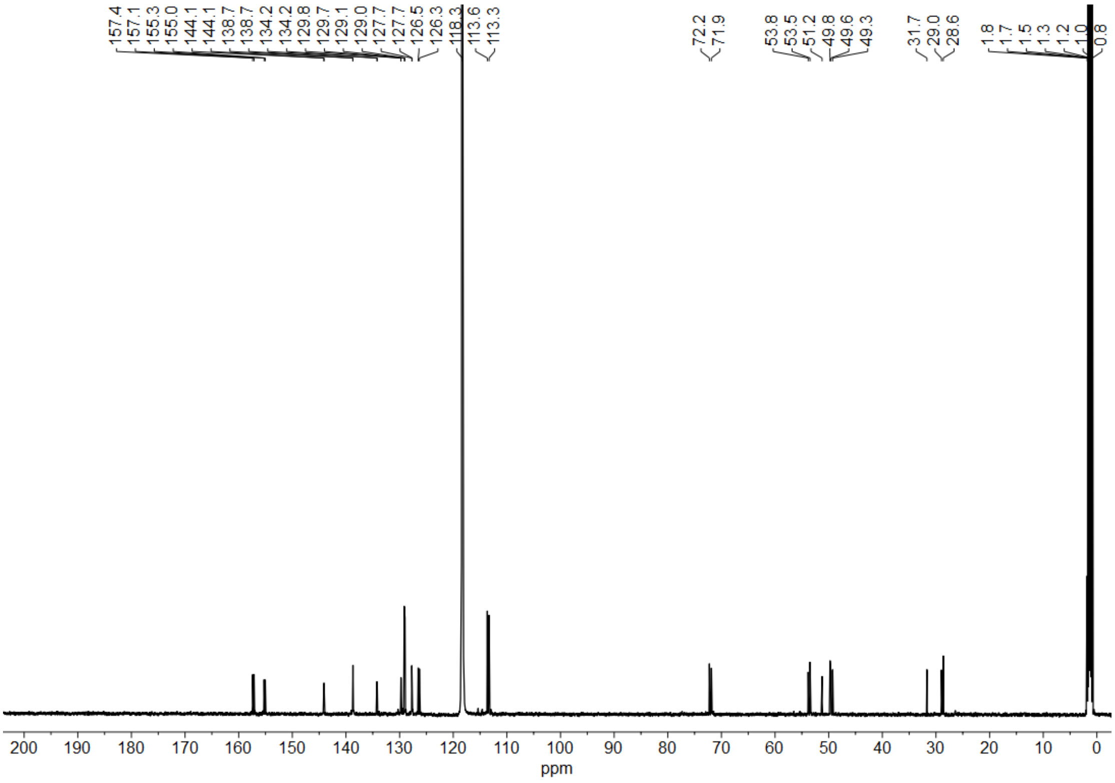

Figure S2. $^{13}C\{^{1}H\}$ NMR (126 MHz, CD_3CN, 298 K) of [Cu(**C$_{6p}$′**)](PF$_6$).

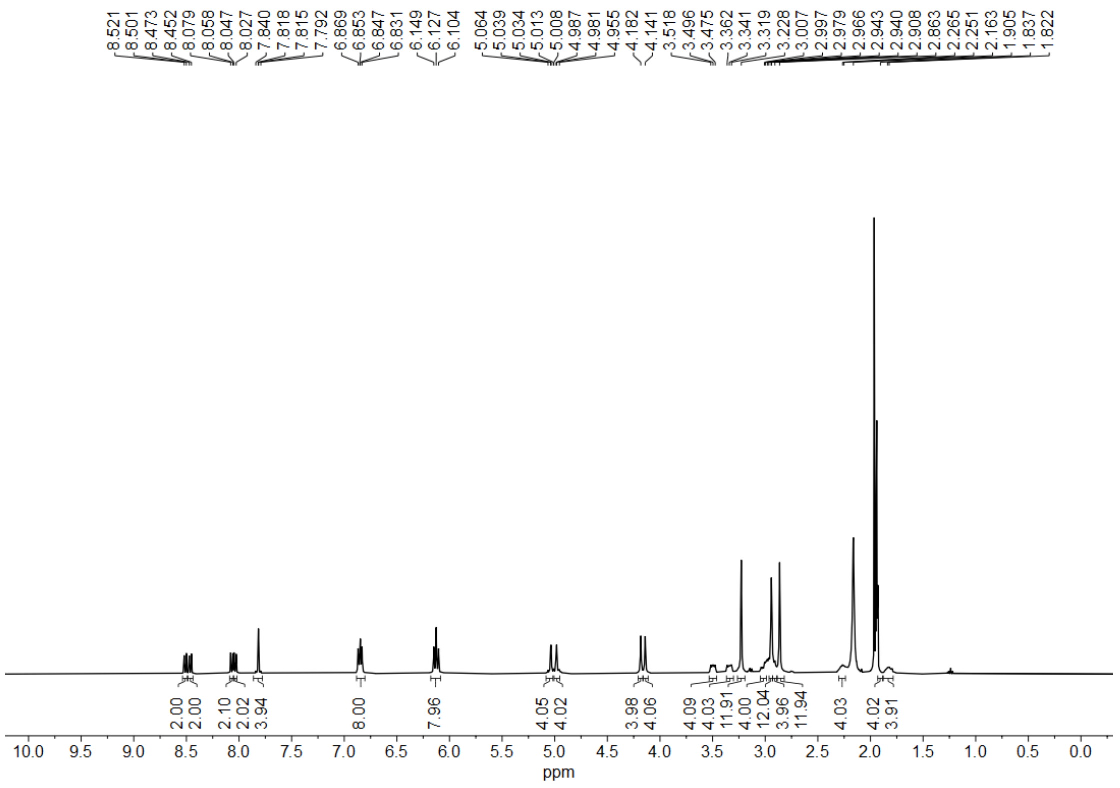

Figure S3. ^{1}H NMR (500 MHz, CD_3CN, 298 K) of [Cu(**C$_{6p}$**)]$(PF_6)_7$.

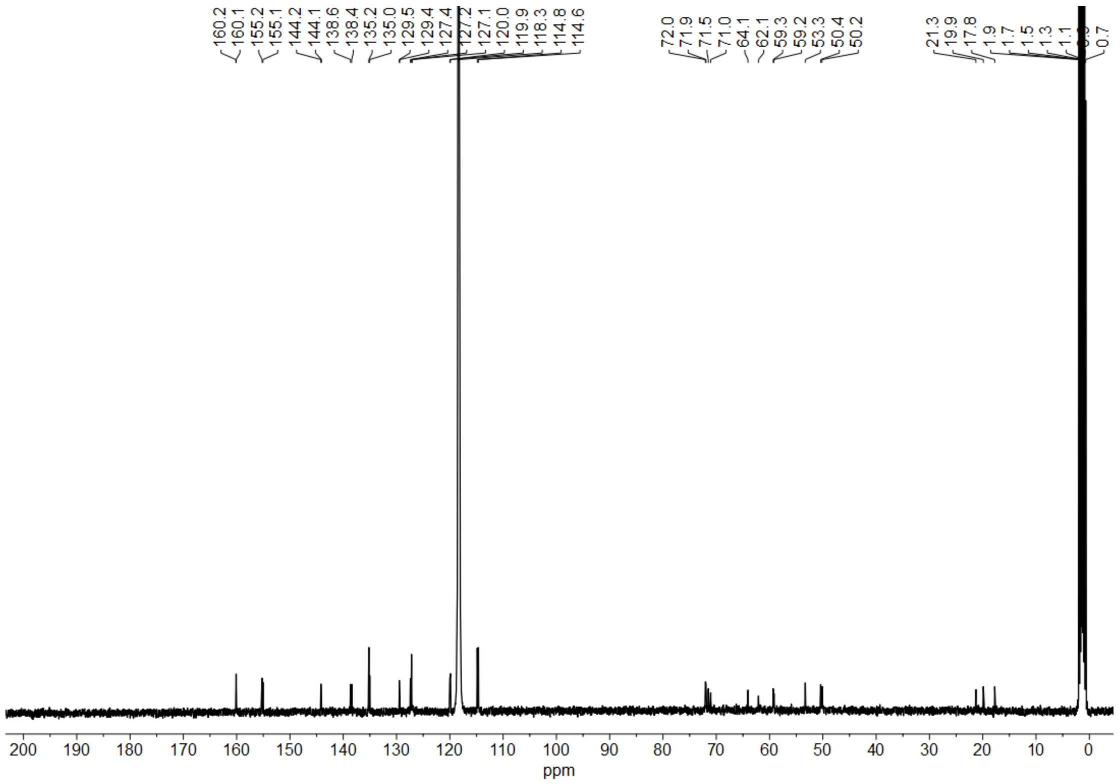

Figure S4. $^{13}C\{^{1}H\}$ NMR (101 MHz, CD_3CN, 298 K) of [Cu(**C$_{6p}$**)]$(PF_6)_7$.

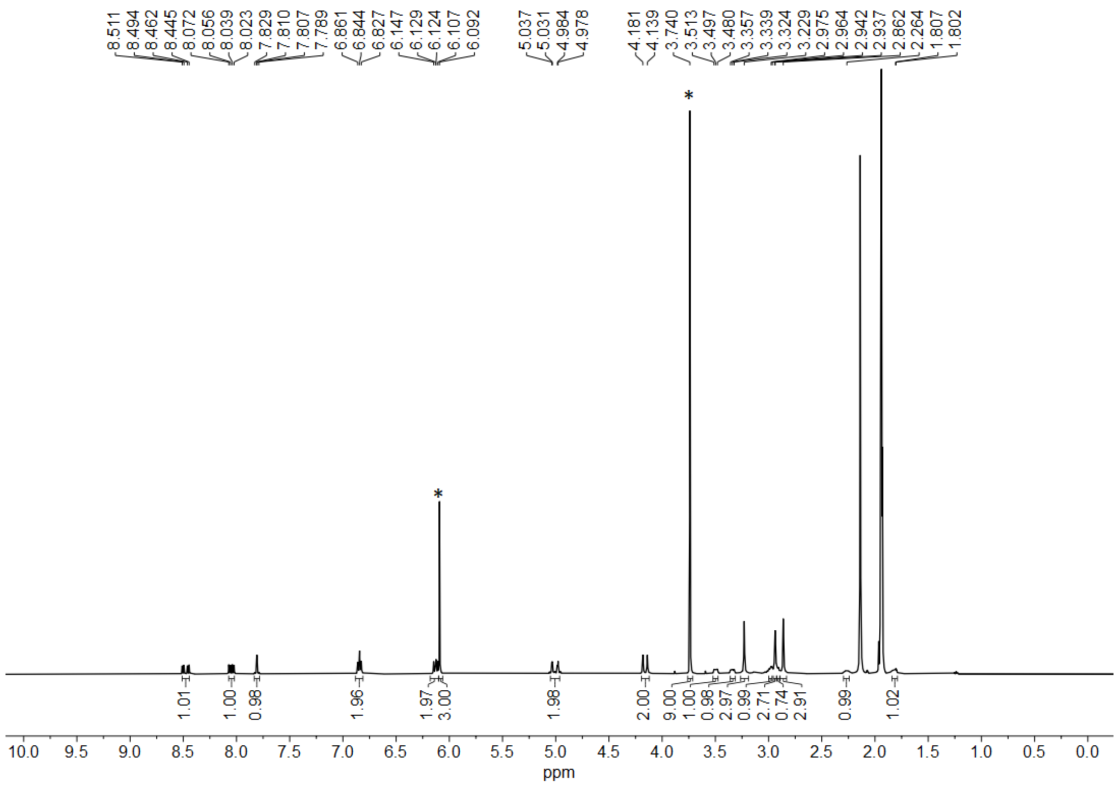

Figure S5. ^{1}H NMR (500 MHz, CD_3CN, 298 K) of $[Cu(\mathbf{C_{6p}})](PF_6)_7$ (1 mM) in the presence of 1,3,5-trimethoxybenzene (marked with *) as an internal standard (2 mM).

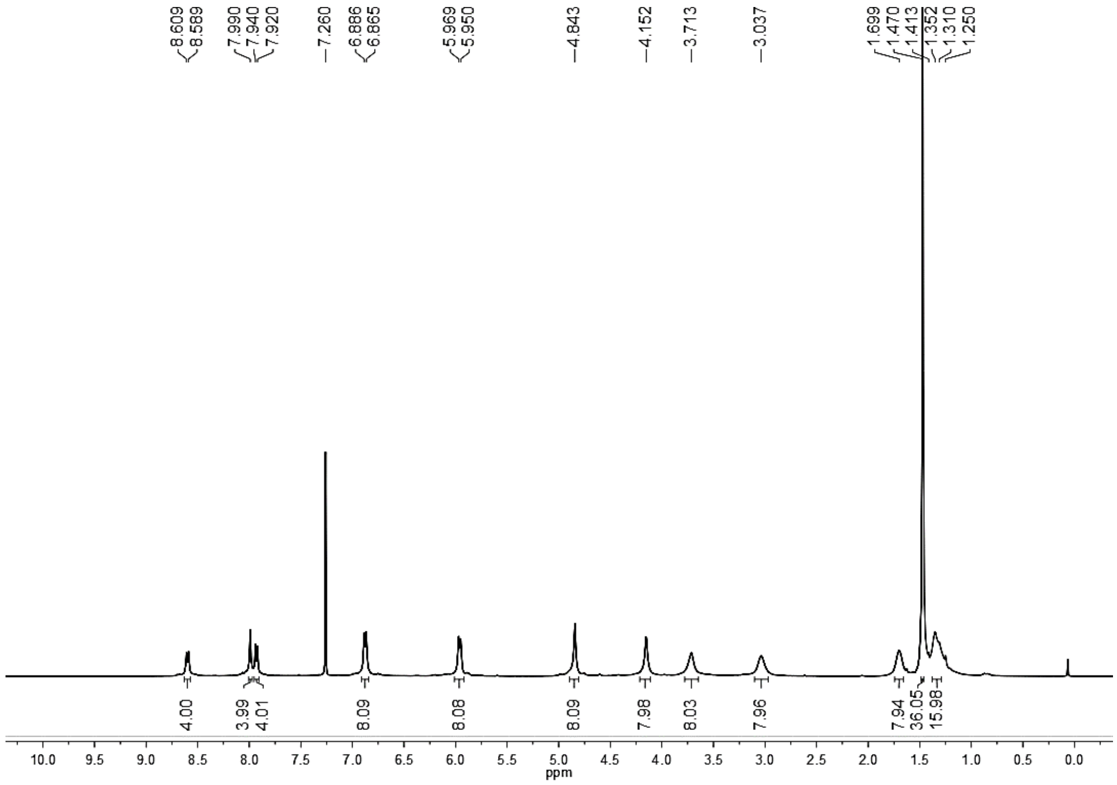

Figure S6. ^{1}H NMR (400 MHz, $CDCl_3$, 298 K) of $[Cu(\mathbf{C_{ester}})](PF_6)$.

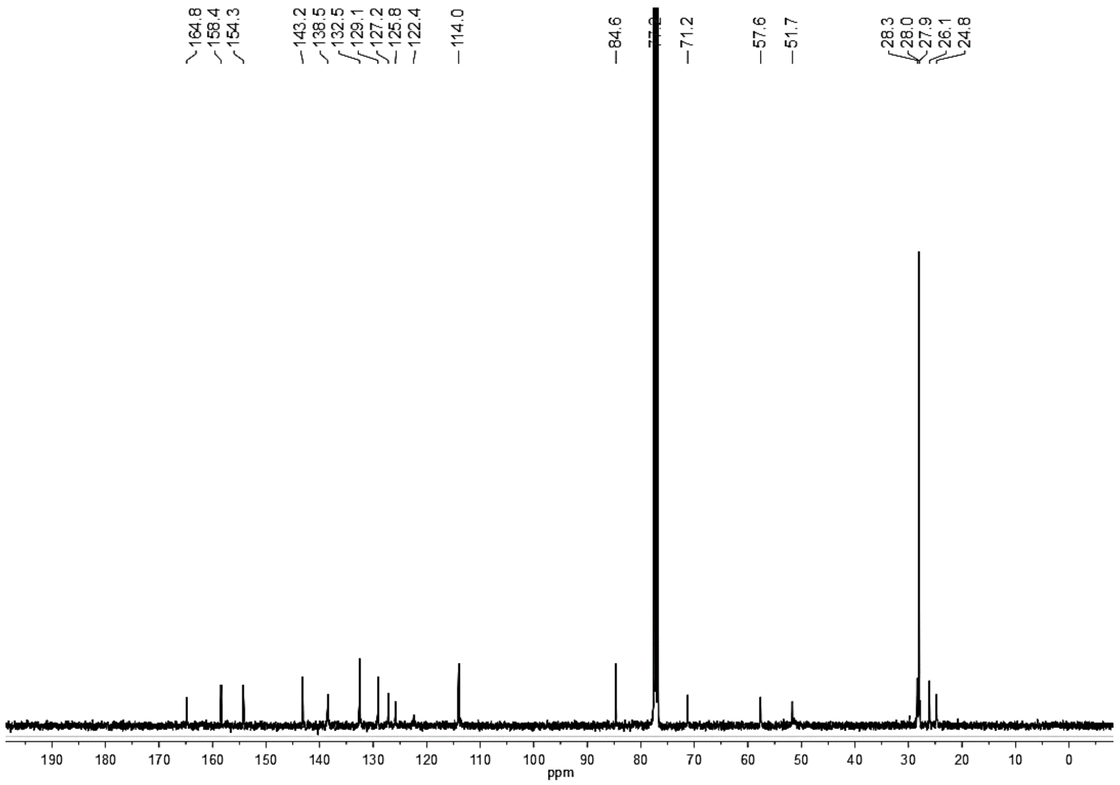

Figure S7. $^{13}C\{^{1}H\}$ NMR (101 MHz, $CDCl_3$, 298 K) of [Cu(**C**$_{\mathbf{ester}}$)](PF_6).

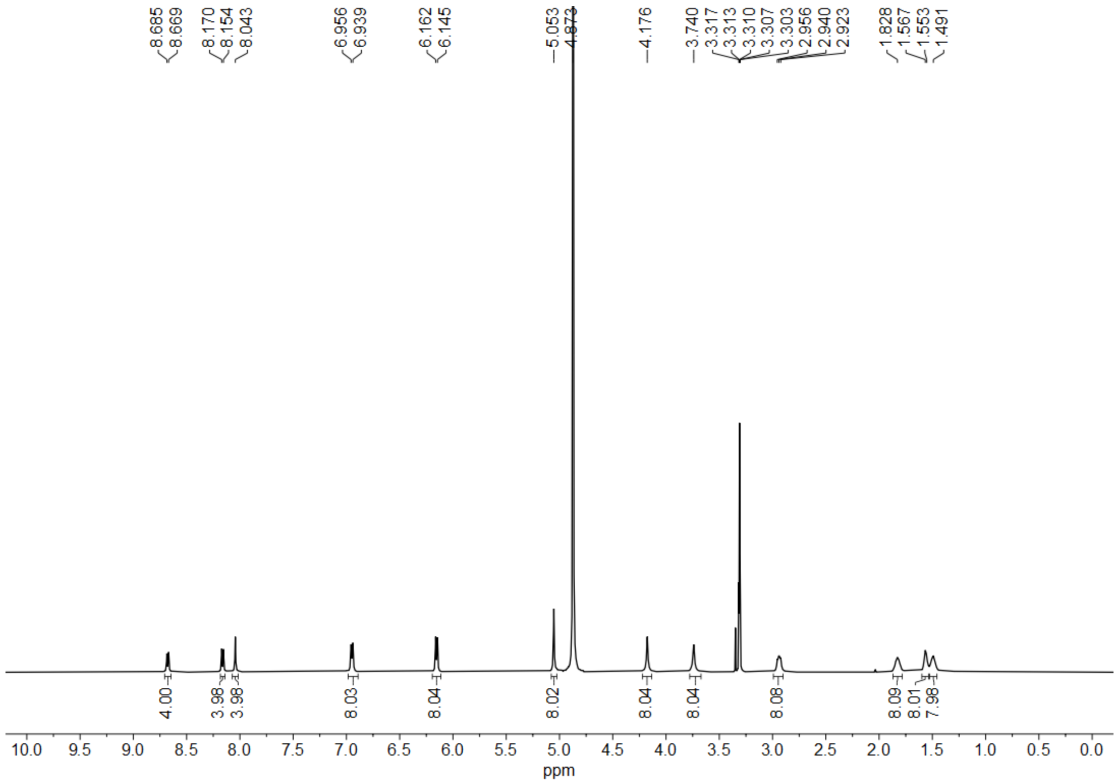

Figure S8. ^{1}H NMR (500 MHz, MeOD, 298 K) of [Cu(**C**$_{\mathbf{4n}}$)](PF_6).

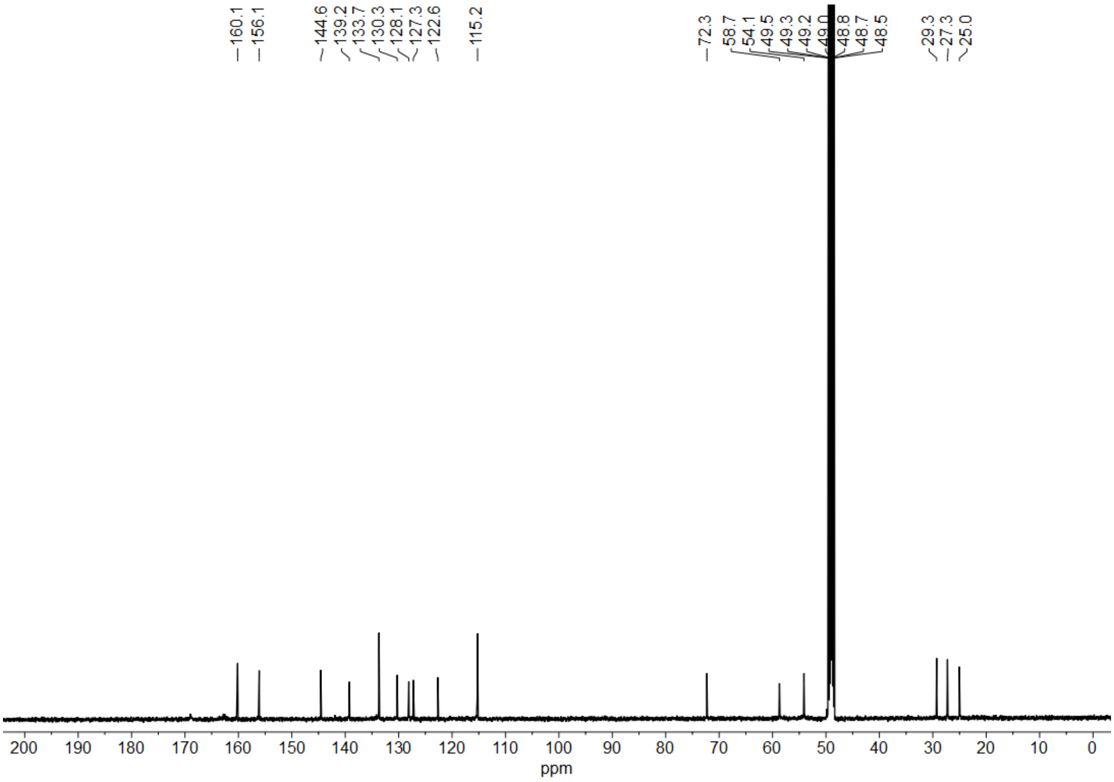

Figure S9. $^{13}C\{^{1}H\}$ NMR (126 MHz, MeOD, 298 K) of [Cu(**C4n**)](PF_6).

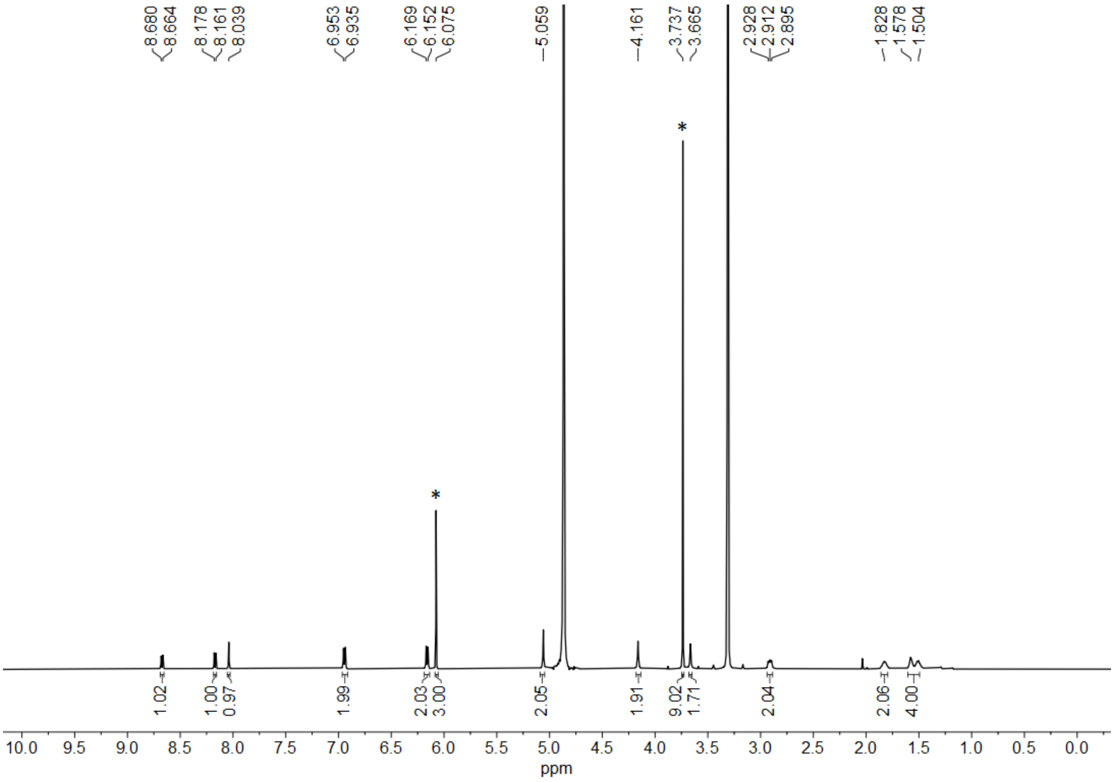

Figure S10. ^{1}H NMR (500 MHz, MeOD, 298 K) of [Cu(**C4n**)](PF_6) (1 mM) in the presence of 1,3,5-trimethoxybenzene (marked with *) as an internal standard (2 mM).

1.3 Mass Spectrometry

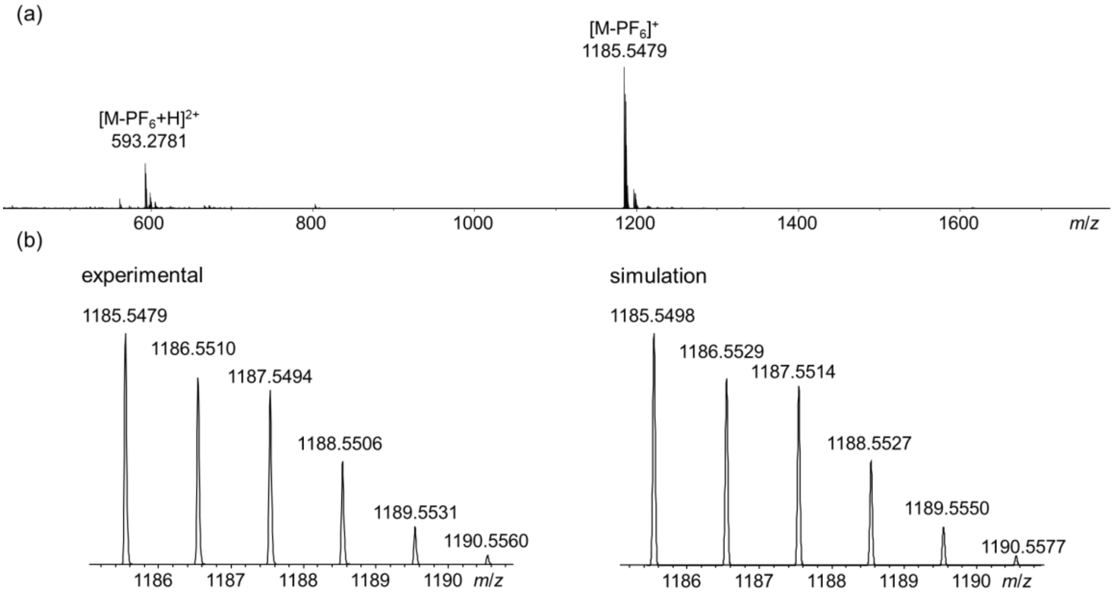

Figure S11. (a) ESI-MS spectrum of [Cu($\mathbf{C_{6p}}'$)](PF_6); (b) HR-ESI-MS spectra of [Cu($\mathbf{C_{6p}}'$)](PF_6) (left: experimental; right: simulation).

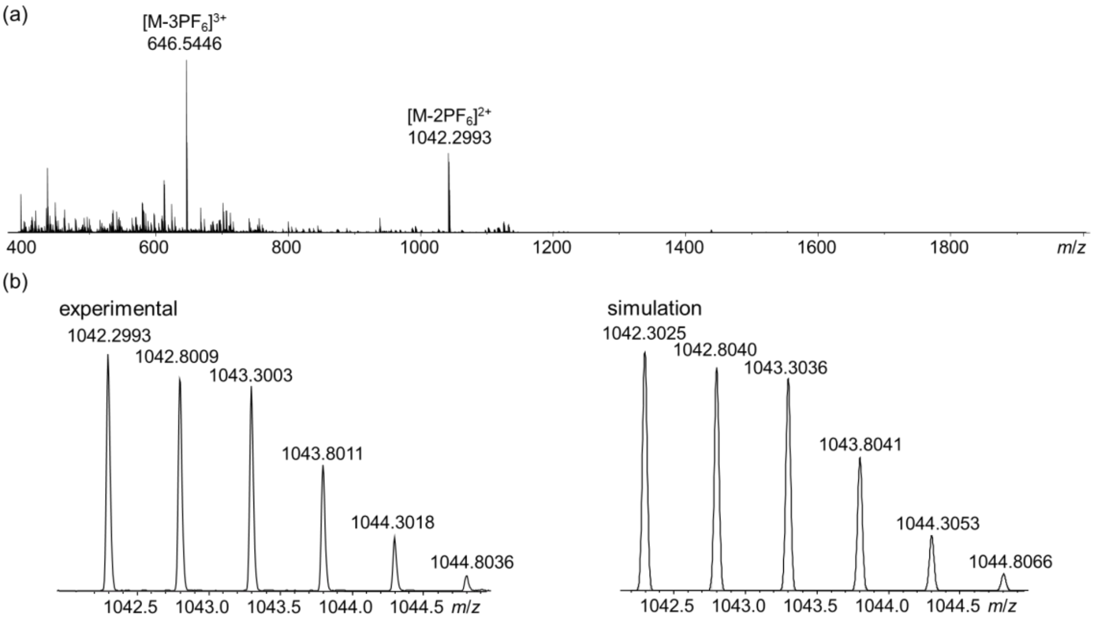

Figure S12. (a) ESI-MS spectrum of [Cu($\mathbf{C_{6p}}$)]$(PF_6)_7$; (b) HR-ESI-MS spectra of [Cu($\mathbf{C_{6p}}$)]$(PF_6)_7$ (left: experimental; right: simulation).

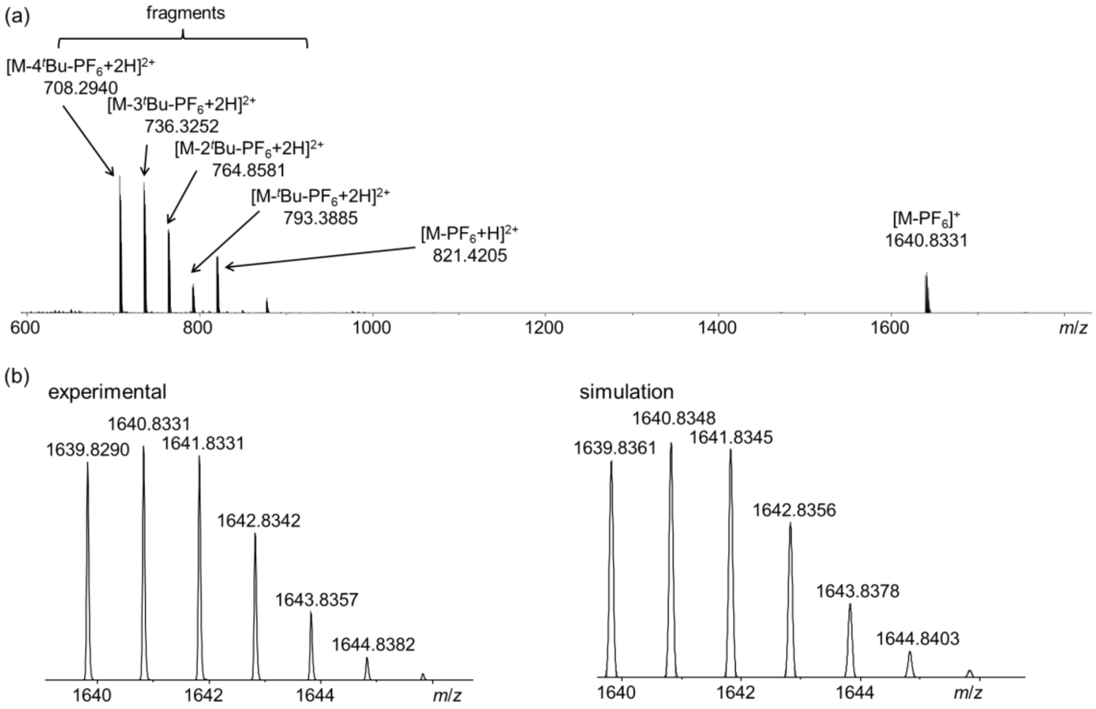

Figure S13. (a) ESI-MS spectrum of [Cu(**C_{ester}**)](PF_6); (b) HR-ESI-MS spectra of [Cu(**C$_{ester}$**)](PF_6). (left: experimental; right: simulation).

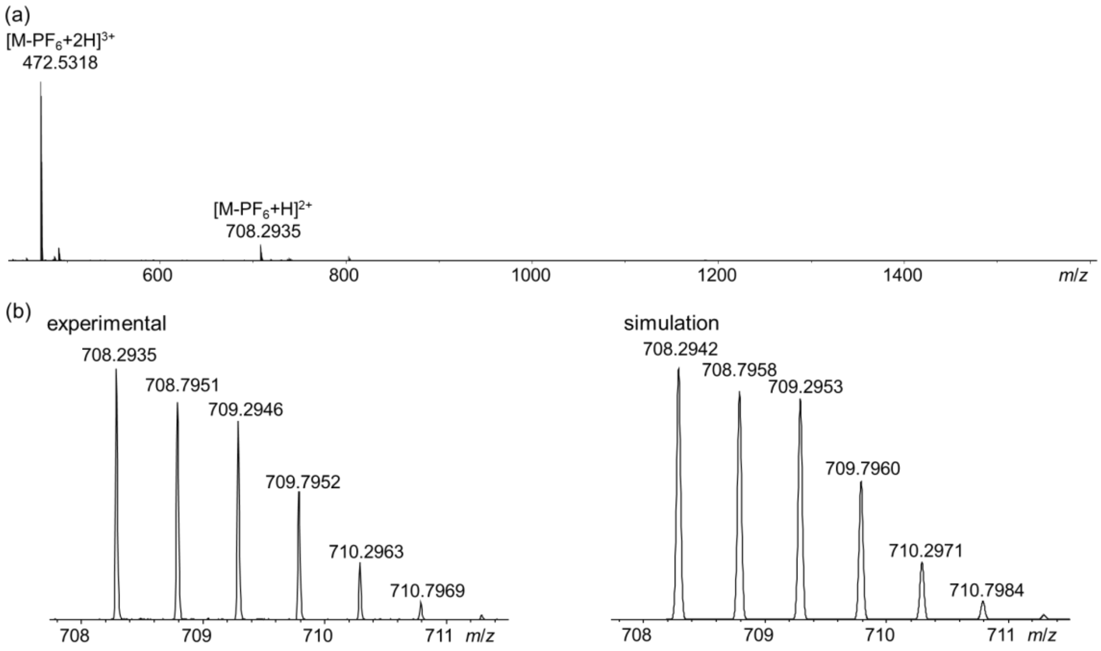

Figure S14. (a) ESI-MS spectrum of [Cu(**C$_{4n}$**)](PF_6); (b) HR-ESI-MS spectra of [Cu(**C$_{4n}$**)](PF_6). (left: experimental; right: simulation).

1.4 UPLC-MS Analysis

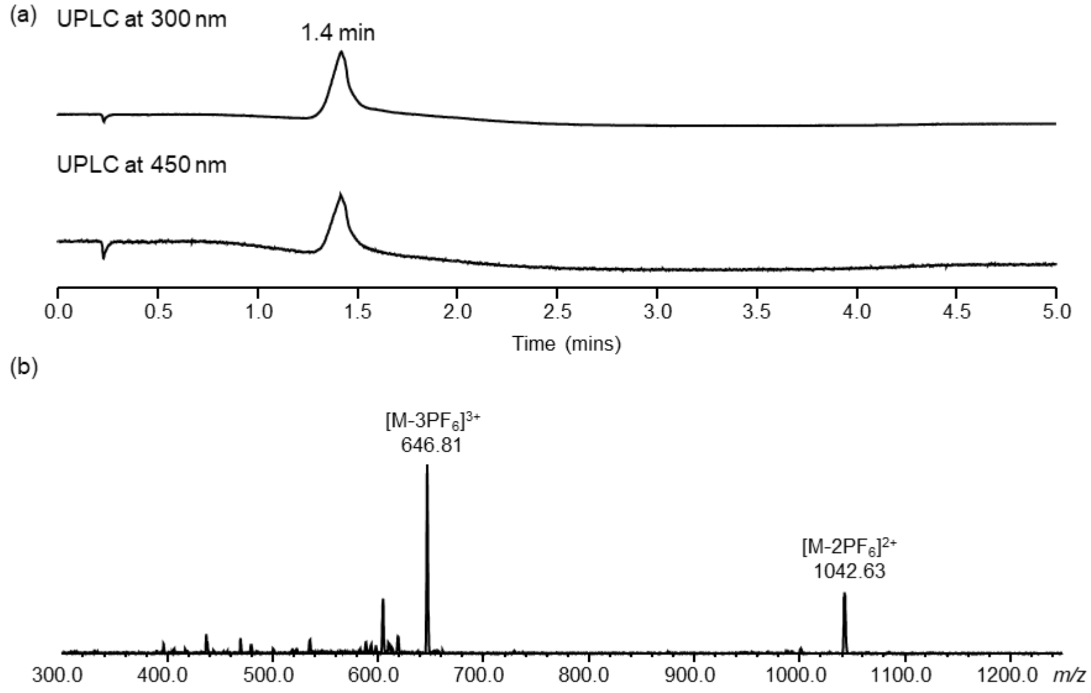

(c) Elution Method (flow rate = 0.5 mL/min)

Time/min	H_2O	MeCN
0.0	95%	5%
3.5	0%	100%
4.5	0%	100%
5.0	95%	5%

Figure S15. UPLC-MS analysis of [Cu($\mathbf{C_{6p}}$)]$(PF_6)_7$. (a) UPLC analysis recorded at 300 nm and 450 nm, respectively; (b) ESI-MS spectrum of the species eluted at 1.4 min; (c) elution method used.

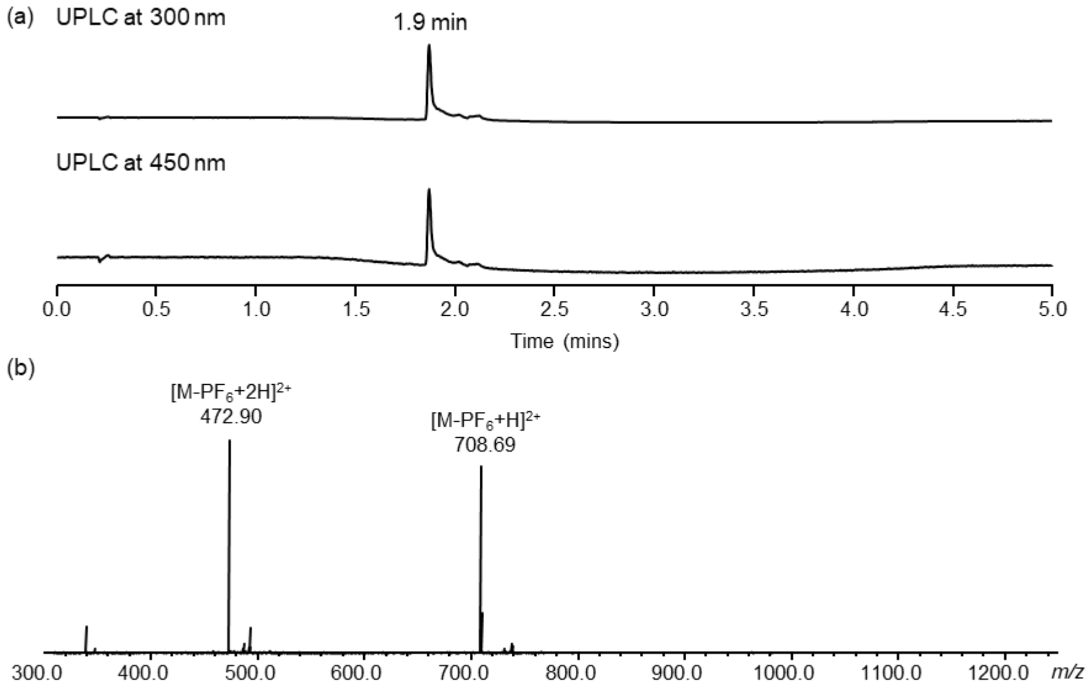

(c) Elution Method (flow rate = 0.5 mL/min)

Time (min)	H_2O (with 0.05% FA)	MeCN (with 0.05% FA)
0.0	95%	5%
0.5	95%	5%
3.5	0%	100%
4.5	0%	100%
5.0	95%	5%

Figure S16. UPLC-MS analysis of [Cu(**C_{4n}**)](PF_6). (a) UPLC analysis recorded at 300 nm and 450 nm, respectively; (b) ESI-MS spectrum of the species eluted at 1.9 min; (c) elution method used.

2. Physical Characterization and Electrochemical Results

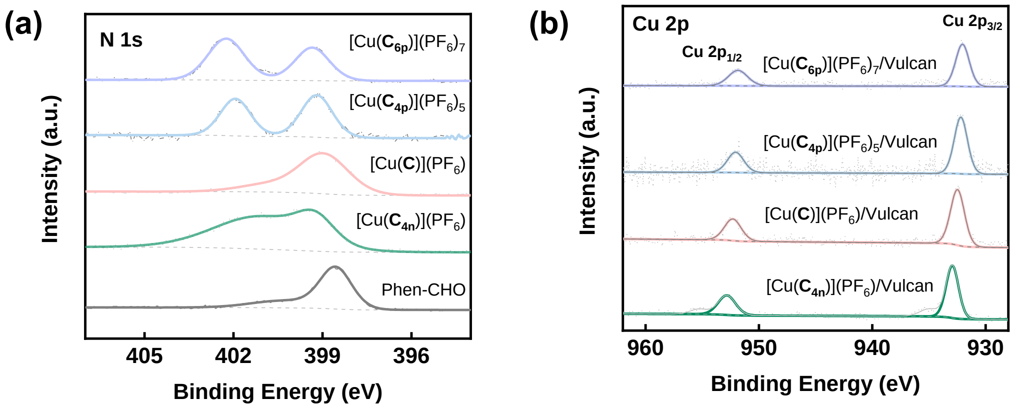

Figure S17. X-ray spectroscopy techniques for Cu centre characterization. (a) High-resolution XPS of N 1s in [Cu(**C_{6p}**)](PF_6)$_7$ (purple), [Cu(**C_{4p}**)](PF_6)$_5$ (blue), [Cu(**C**)](PF_6) (pink), [Cu(**C_{4n}**)](PF_6) (green), and **Phen-CHO** (grey); (b) High-resolution XPS of Cu 2p in [Cu(**C_{6p}**)](PF_6)$_7$ (purple), [Cu(**C_{4p}**)](PF_6)$_5$ (blue), [Cu(**C**)](PF_6) (pink), and [Cu(**C_{4n}**)](PF_6) (green) supported on Vulcan.

In the N 1s spectra of **Phen-CHO** and [Cu(**C**)](PF_6), similar features with the peaks at 399 eV are observed, assignable to the N species of the phenanthroline ligand.[4] Similar binding energy peaks at *ca.* 399 eV were found for [Cu(**C_{6p}**)](PF_6)$_7$, [Cu(**C_{4p}**)](PF_6)$_5$, [Cu(**C**)](PF_6), and [Cu(**C_{4n}**)](PF_6), which are slightly shifted towards a higher binding energy by 1-1.5 eV when compared to that of the metal-free phenanthroline ligand **Phen-CHO**, attributing to the Cu-N coordination in the Cu(I) catenanes. Additional peaks at the binding energy of *ca.* 402 eV in the spectra of [Cu(**C_{6p}**)](PF_6)$_7$, [Cu(**C_{4p}**)](PF_6)$_5$ are assigned to the N species of the cationic ammonium groups in the catenanes (Figure S17a).[5]

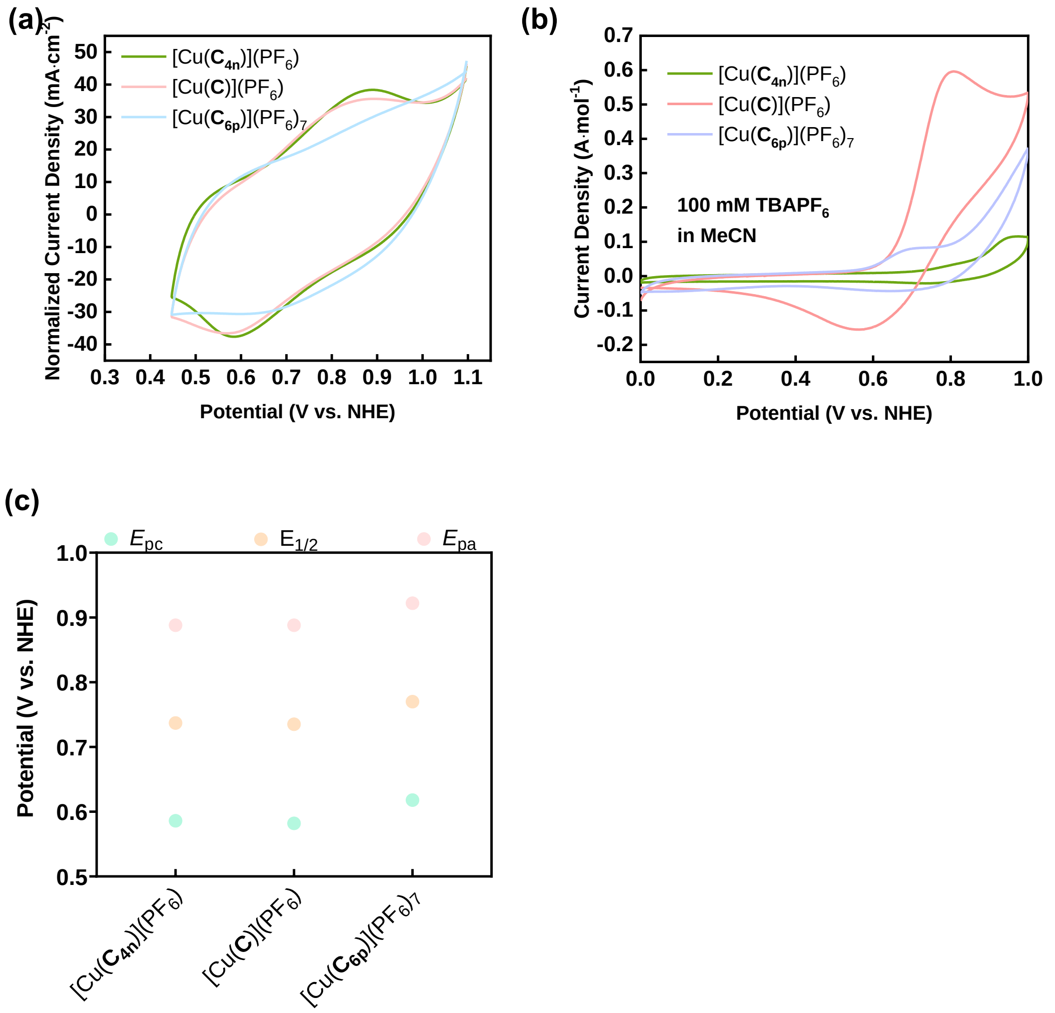

Figure S18. Cyclic voltammograms (CVs) of [Cu(**C$_{4n}$**)](PF$_6$) (green), [Cu(**C**)](PF$_6$) (pink), and [Cu(**C$_{6p}$**)](PF$_6$)$_7$ (blue) collected (a) on Vulcan with a scan rate of 50 mV/s in N_2-saturated 5x PBS buffer at pH 7; (b) with unsupported complexes in N_2-saturated acetonitrile with 100 mM (^{n}Bu$_4$N)(PF$_6$); (c) E_{pa} (pink), $E_{1/2}$ (orange), and E_{pc} (green) of [Cu(**C$_{4n}$**)](PF$_6$), [Cu(**C**)](PF$_6$), and [Cu(**C$_{6p}$**)](PF$_6$)$_7$ measured on Vulcan.

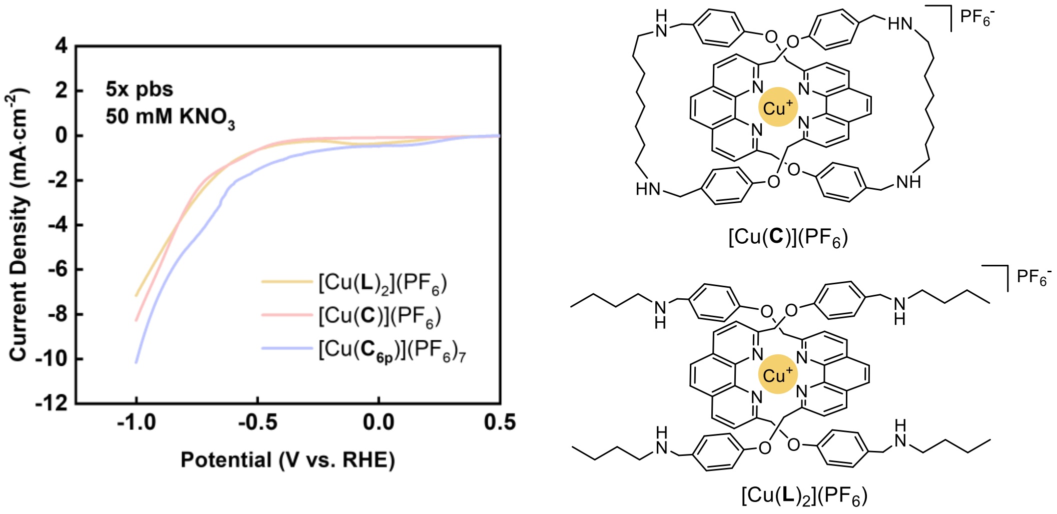

Figure S19. Nitrate reduction LSVs of [Cu(**L**)$_2$](PF_6) (yellow), [Cu(**C**)](PF_6) (pink), and [Cu(**C$_{6p}$**)](PF_6)$_7$ (purple) in Ar-saturated 5x PBS solution with 50 mM KNO_3.

The non-interlocked Cu(I) complex [Cu(**L**)$_2$](PF_6), supported by phenanthroline ligands of a similar chemical composition as [Cu(**C**)](PF_6), was further studied for its nitrate reduction performance. Only a small difference in the onset potential was found when comparing [Cu(**C**)](PF_6) (-0.43 V vs. RHE) with [Cu(**L**)$_2$](PF_6) (-0.45 V vs. RHE) (Figure S19), suggesting that a simple interlocking of the ligand may not have a significant effect on the NO_3RR activity of the copper. Yet, the mechanical interlocking in the catenane is essential to the installation of the cationic ammoniums on the ligand backbones, which are necessary for the enhanced NO_3RR activity and selectivity as discussed in the main text. In the absence of mechanical interlocking, Coulombic repulsion between cationic ammoniums cannot be fully compensated by the Cu(I)-phenanthroline coordination, and hence structural integrity of the Cu(I) bis(phenanthroline) cannot be maintained. In fact, synthesis and isolation of a non-interlocked analogue of [Cu(**C$_{4p}$**)](PF_6)$_5$ was unsuccessful,[3] and NMR and MS studies indicated the presence of a dynamic mixture consisting of Cu(I) complexes of the dicationic phenanthroline in various stoichiometry. An even stronger ligand-ligand repulsion is therefore expected if additional positive charges are to be incorporated into the ligand framework.

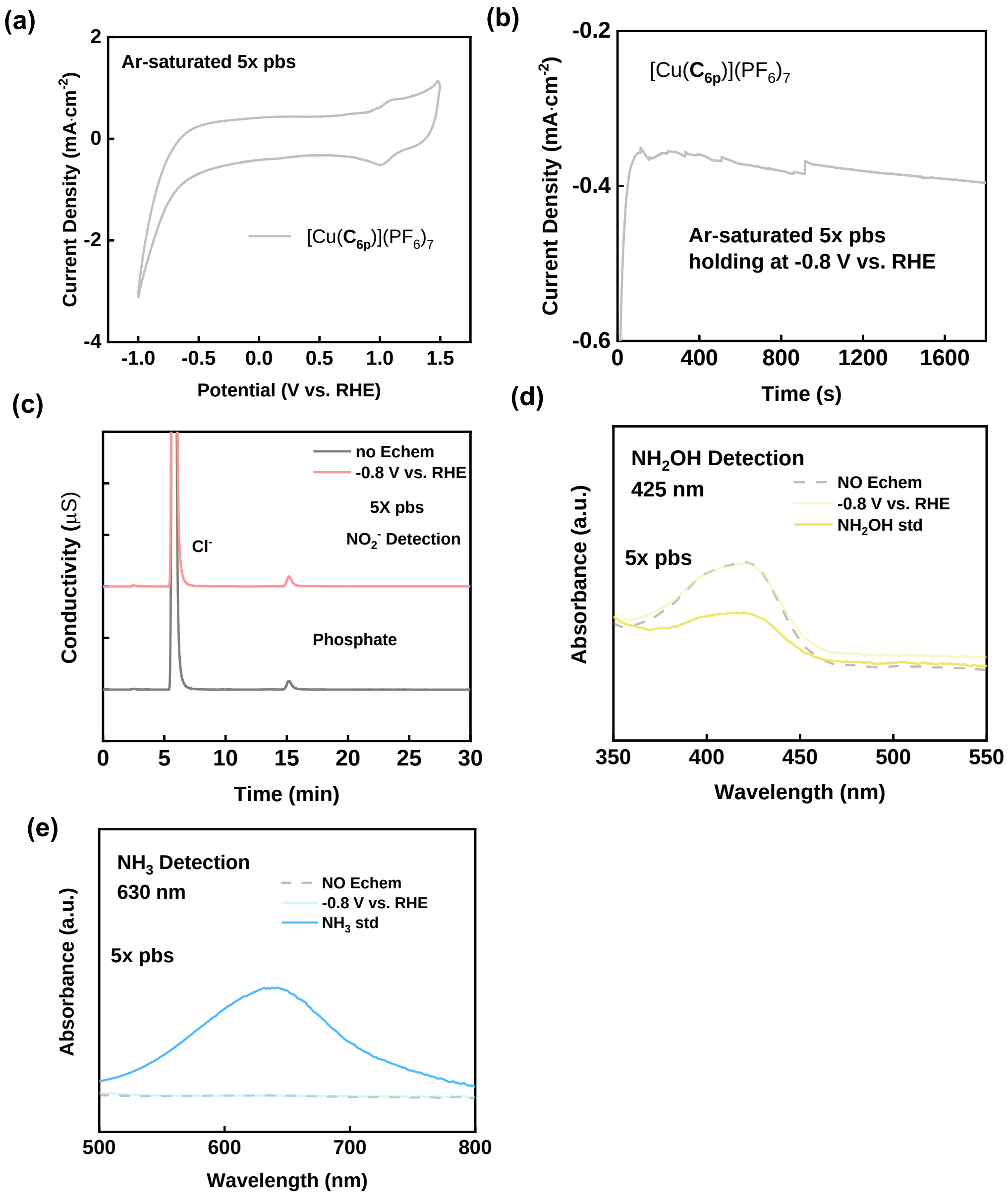

Figure S20. (a) Cyclic voltammograms (CVs) of [Cu(**C6p**)](PF_6)$_7$ in Ar-saturated and NO_3^--free 5x PBS buffer. (b) Chronoamperometry at -0.8 V vs. RHE for 30 min. Product analysis after 30 min electrolysis of [Cu(**C6p**)](PF_6)$_7$ in Ar-saturated and NO_3^--free 5x PBS buffer employing (c) ion chromatography for detecting NO_2^-; (d) UV-Vis spectroscopy for detecting NH_2OH; (e) UV-Vis spectroscopy for detecting NH_3.

To confirm that the added nitrate is the sole N-atom source, electrochemical studies were conducted under argon in the absence of added nitrate. No catalytic current at the cathodic potential for NO_3RR was recorded, and no NO_2^-, NH_2OH, nor NH_3 was identified (Figure S20). These findings consistently support that nitrate is the sole nitrogen source in the NO_3RR.

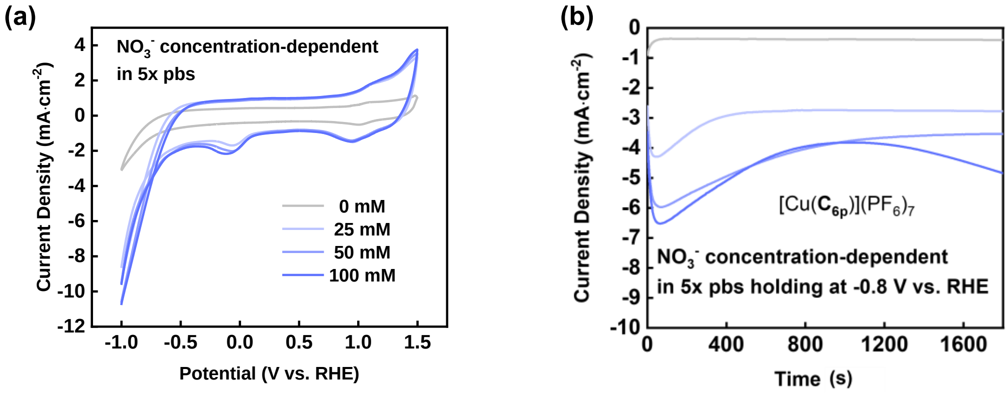

Figure S21. (a) Cyclic voltammograms (CVs) of [Cu(**C_{6p}**)](PF_6)$_7$ in the presence of different concentrations of NO_3^-. (b) Chronoamperometry at -0.8 V vs. RHE for 30 min in the presence of different concentrations of NO_3^-.

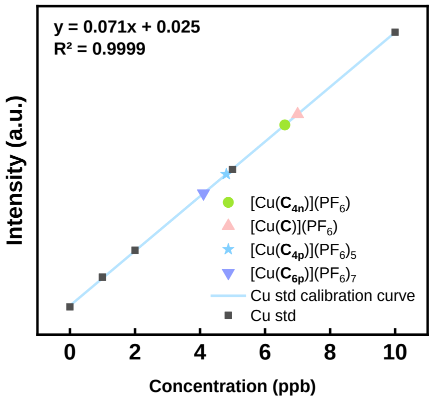

Figure S22. ICP-MS calibration curve of Cu for [Cu(**C_{4n}**)](PF_6) (green), [Cu(**C**)](PF_6) (pink), [Cu(**C_{4p}**)](PF_6)$_5$ (blue), and [Cu(**C_{6p}**)](PF_6)$_7$ (purple).

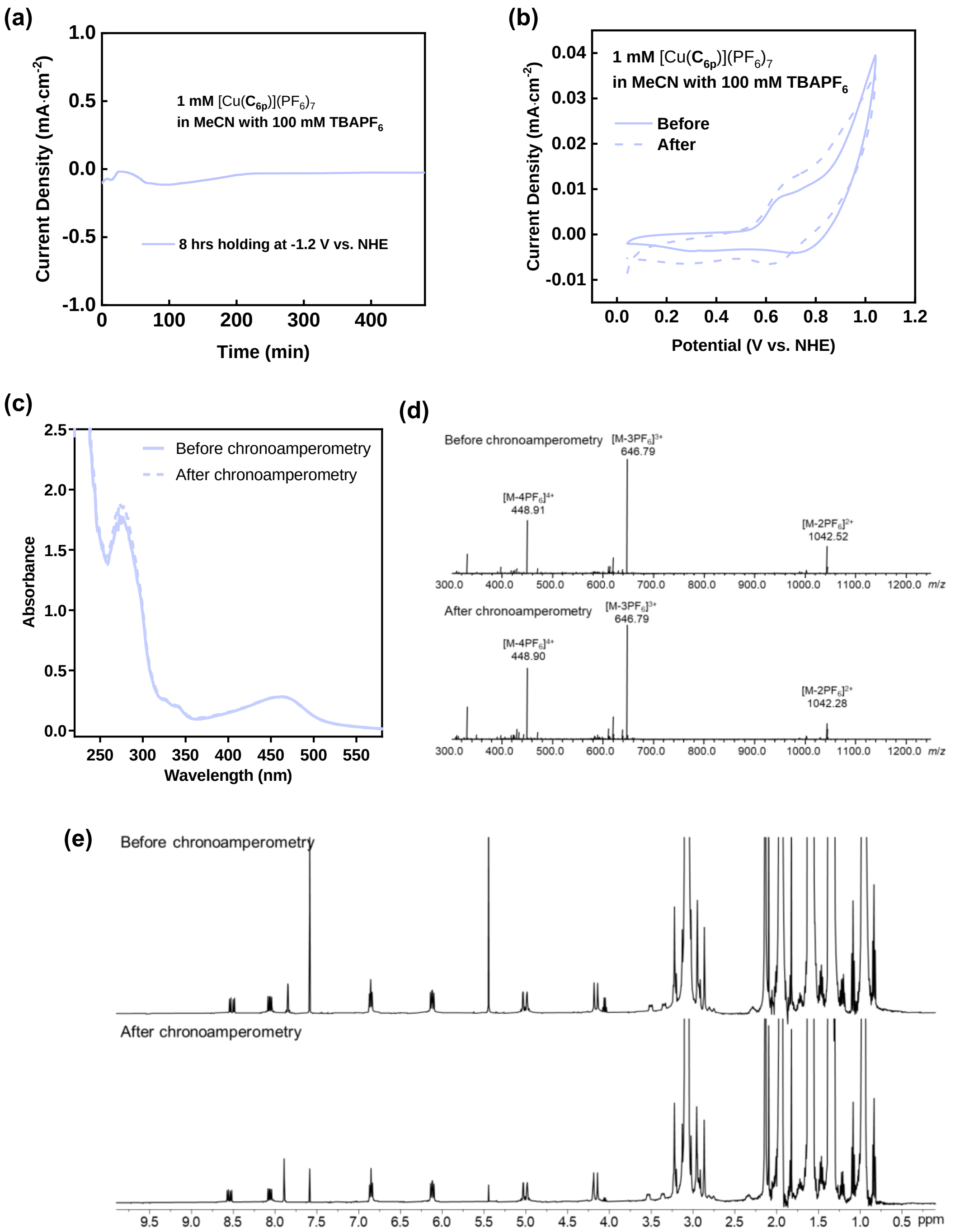

Figure S23. (a) Chronoamperometry of the 1 mM solution of [Cu(**C6p**)]$(PF_6)_7$ in MeCN containing 100 mM of the electrolyte $(^{n}Bu_4N)(PF_6)$ at -1.2 V vs. NHE for 8 hours; (b) Cyclic voltammograms (CVs) before (solid) and after (dash) 8 hours holding at -1.2 V vs. NHE, with a scan rate of 50 mV/s recorded in N_2-saturated acetonitrile with 100 mM $N^{n}Bu_4PF_6$; (c) UV-Vis spectra of [Cu(**C6p**)]$(PF_6)_7$ before (solid) and after (dash) 8 hours holding at -1.2 V vs. NHE; (d) ESI-MS analysis, and (e) ^{1}H NMR spectra (500 MHz, CD_3CN, 298 K) of [Cu(**C6p**)]$(PF_6)_7$ before (top) and after (bottom) 8 hours holding at -1.2 V vs. NHE.

3. Product Analysis

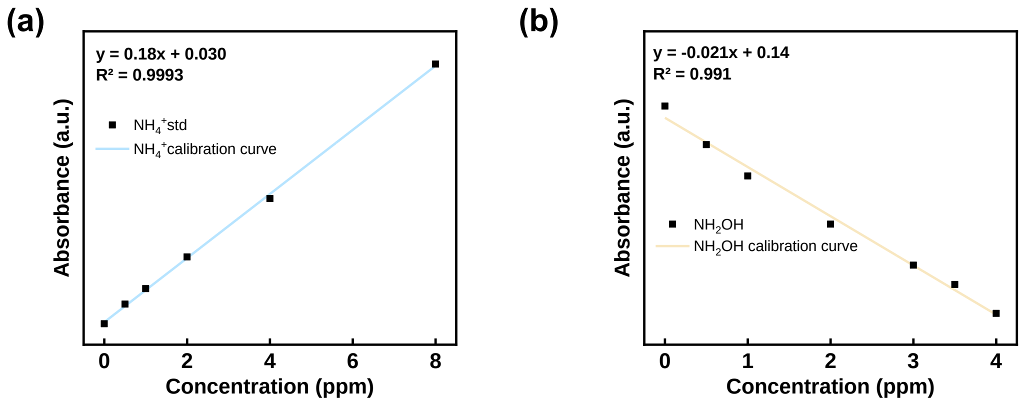

Figure S24. Calibration curves for (a) NH_3 (λ = 630 nm) and (b) NH_2OH (λ = 425 nm).

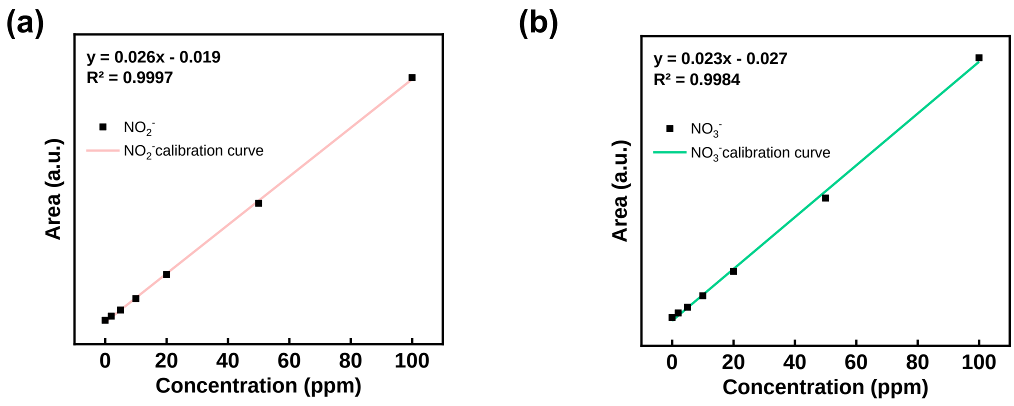

Figure S25. Calibration curves for (a) NO_2^- and (b) NO_3^- quantified by ion chromatography (IC) with an electrochemical detector (ECD).

4. Kinetic Isotope Effect Studies

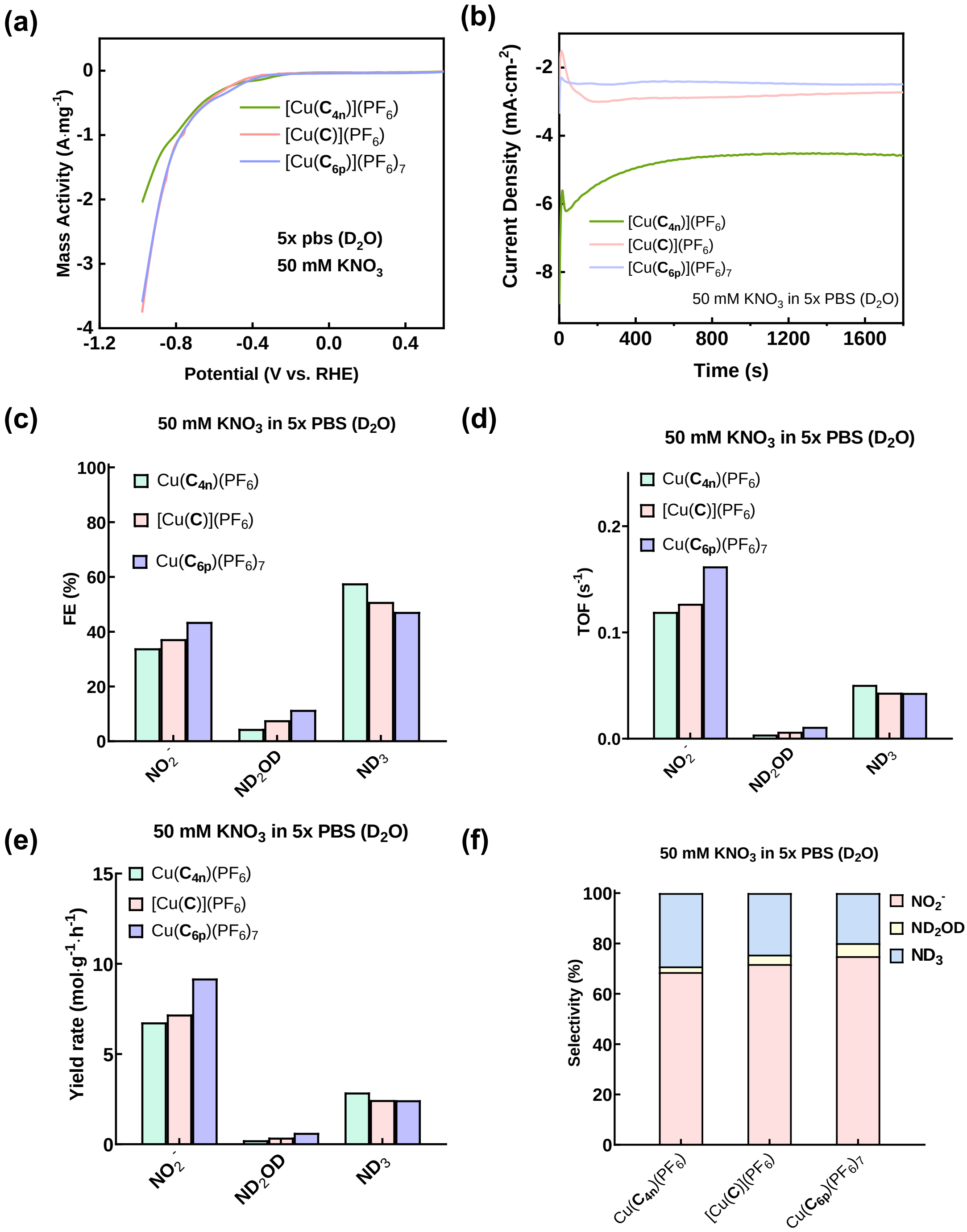

Figure S26. Kinetic isotope effect. (a) Nitrate reduction LSVs of [Cu(**C$_{4n}$**)](PF$_6$) (green), [Cu(**C**)](PF$_6$) (pink), and [Cu(**C$_{6p}$**)](PF$_6$)$_7$ (blue) in Ar-saturated deutero 5x PBS solution with 50 mM KNO_3; (b) Chronoamperometry at -0.8 V vs. RHE for 30 min; (c) Faradic efficiency, (d) turnover frequency, and (e) yield rate of NH_3, NH_2OH, and NO_2^- of [Cu(**C$_{4n}$**)](PF$_6$) (green), [Cu(**C**)](PF$_6$) (pink), and [Cu(**C$_{6p}$**)](PF$_6$)$_7$ (blue) at -0.8 V vs. RHE in Ar-saturated deutero 5x PBS solution with 50 mM KNO_3; (f) Product selectivity of [Cu(**C$_{4n}$**)](PF$_6$), [Cu(**C**)](PF$_6$), and [Cu(**C$_{6p}$**)](PF$_6$)$_7$ at -0.8 V vs. RHE in Ar-saturated deutero 5x PBS solution with 50 mM KNO_3.

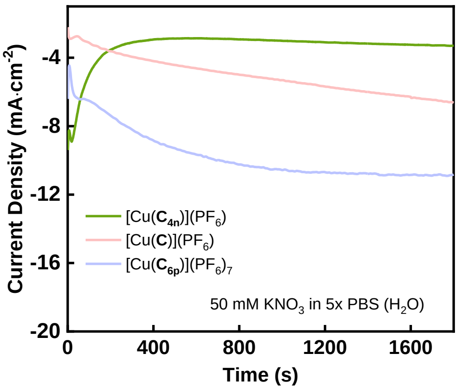

Figure S27. Chronoamperometry of [Cu($\mathbf{C_{4n}}$)](PF_6) (green), [Cu(**C**)](PF_6) (pink), and [Cu($\mathbf{C_{6p}}$)](PF_6)$_7$ (blue) in Ar-saturated proteo 5x PBS solution with 50 mM KNO_3 at -0.8 V vs. RHE nitrate reduction for 30 min.

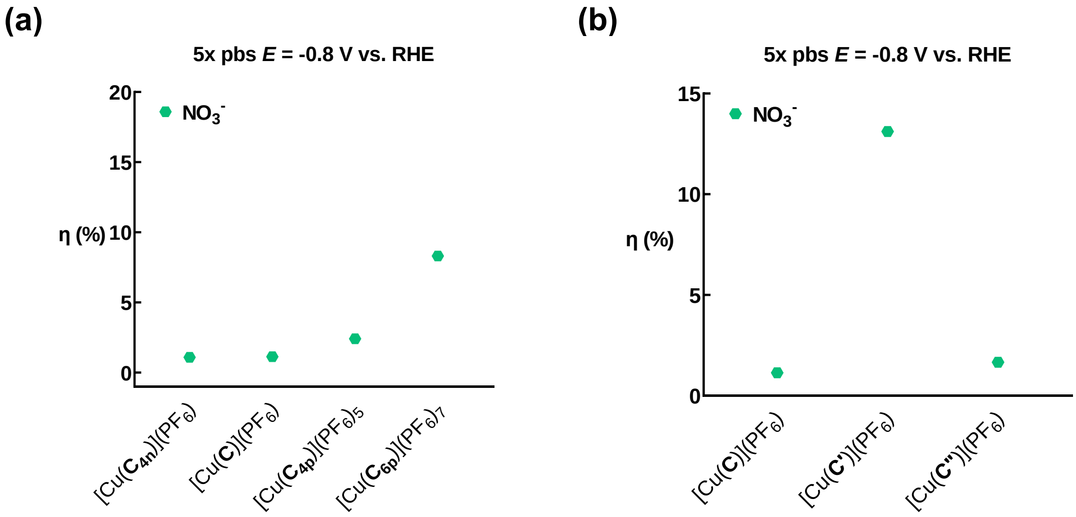

Figure S28. NO_3^- conversion rates of (a) [Cu($\mathbf{C_{4n}}$)](PF_6), [Cu(**C**)](PF_6), and [Cu($\mathbf{C_{6p}}$)](PF_6)$_7$; and (b) [Cu(**C**)](PF_6), [Cu(**C′**)](PF_6), and [Cu(**C″**)](PF_6).

Table S1. List of FE and yield rate of state-of-the-art copper-based catalysts for NO_3RR to generate NH_3.

Catalyst	FE (%)	Yield rate	Electrolyte	Condition	pH	E vs. RHE	Ref.
			Molecular Complex				
[Cu(**C$_{6p}$**)]$(PF_6)_7$/Vulcan	86	8500 mmol g^{-1}_{Cu} h^{-1}	5x PBS	50 mM KNO_3	7	-0.80	This work
[Cu(**C′**)](PF_6)/Vulcan	95	17000 mmol g^{-1}_{Cu} h^{-1}	5x PBS	50 mM KNO_3	7	-0.80	This work
CuF16Pc/C	78	125 mmol g^{-1}_{Cu} h^{-1}	50 mM Na_2SO_4	80 mM NO_3^-	7	-0.90	[6]
(CuPc) MDE	98	17000 mmol g^{-1}_{Cu} h^{-1}	1000 mM KOH	1000 mM KNO_3	14	-0.32	[7]
CuPc-GCE	64	N/A	100 mM KOH	50 mM KNO_3	13	-0.89	[8]
CoQPyPhenI	94	505 mmol g^{-1}_{Co} h^{-1}	100 mM K_2SO_4	100 mM KNO_3	7	-0.60	[9]
			Copper Single Atom				
Cu(I)–N_3C_1	52	5466 mmol g^{-1}_{Cu} h^{-1}	50 mM Na_2SO_4	7.14 mM $NaNO_3$	7	-0.64	[10]
Cu-N-C SAC	84	12500 mmol g^{-1}_{Cu} h^{-1}	100 mM KOH	100 mM KNO_3	13	-1.00	[11]
Cu-N-C	94	542 mmol g^{-1}_{Cu} h^{-1}	500 mM Na_2SO_4	3.6 mM $NaNO_3$	7	-0.85	[12]
Cu-*cis*-N_2O_2	88	1.69 mmol cm^{-2} h^{-1}	500 mM Na_2SO_4	1000 mg mL^{-1} N–KNO_3	7	-1.60	[13]
Cu-NC	82	278 mmol g^{-1}_{Cu} h^{-1}	200 mM Na_2SO_4	500 mM $NaNO_3$	7	-0.89	[14]
			Nanostructured Copper and Supported Copper				
Cu disc	68	N/A	1000 mM $HClO_4$	5 mM NO_3^-	0	-0.31	[15]
Cu nanodisk	81	127 mmol g^{-1}_{Cu} h^{-1}	100 mM KOH	10 mM KNO_3	13	-0.50	[16]
Cu nanosheet	99	23 mmol g^{-1}_{Cu} h^{-1}	100 mM KOH	10 mM KNO_3	13	-0.15	[17]
$Ag_{20}Cu_{12}$ nanocluster	85	1380 mmol g^{-1}_{cat} h^{-1}	500 mM Na_2SO_4	8 mM $NaNO_3$	7	-0.60	[18]
MOFs@CuPc	94	88.2 mmol g^{-1}_{cat} h^{-1}	500 mM Na_2SO_4	0.5 mM $NaNO_3$	7	-0.75	[19]
**1**-Cu	86	3137 mmol g^{-1}_{Cu} h^{-1}	500 Na_2SO_4	5 mM $NaNO_3$	7	-0.9	[20]
NiBDC@HsGDY@Cu	99	6230 mmol cm^{-2} h^{-1}	1000 mM KOH	100 mM KNO_3	14	-0.41	[21]

			Copper Oxide				
Cu@Cu2+1O NWs	78	33.8 mmol g^{-1}_{cat} h^{-1}	500 K_2SO_4	7.1 mM $NaNO_3$	7	-0.55	[22]
plasma-treated Cu_2O	89	0.08 mmol cm^{-2} h^{-1}	500 mM Na_2SO_4	3.2 mM $NaNO_3$	7	-0.49	[23]
Cu_2O	98	N/A	500 mM Na_2SO_4	5 mM $NaNO_3$	7	-0.80	[24]
Cu_2O(100) Facets	82	43.6 mmol g^{-1}_{cat} h^{-1}	100 mM Na_2SO_4	3.6 mM $NaNO_3$	7	-0.60	[25]

Table S1 shows the FE and yield rate of ammonia generated from NO_3RR catalysed by the copper catenane complexes reported in this study as well as copper catalysts in various forms reported in previous literature. The reported copper catenane complexes exhibit much higher yield rates and desirable FE when compared with other reported molecular copper complexes and various copper-based materials.

5. Computational Investigation

Density Functional Theory (DFT) Calculation Methods

Density Functional Theory (DFT) calculations were conducted to investigate the deprotonation state of [Cu(**C4n**)](PF_6). The studies involved five distinct protonation states with different numbers of carboxylic acids (RCOOH) and carboxylates ($RCOO^-$): M^+, [M-H], $[M\text{-}2H]^-$, $[M\text{-}3H]^{2-}$, $[M\text{-}4H]^{3-}$ (M = [Cu(**C4n**)]), using Avogadro software.[26] These structures were subsequently optimized through DFT calculations with Gaussian 16 software package.[27]

Geometry optimizations and single-point energy calculations for all structures utilized the spin-unrestricted hybrid density functional B3LYP.[28-30] Consistent with prior studies on copper complexes, a mixed basis set was applied, employing LANL2DZ for copper and 6-31G(d,p) for the remaining atoms.[31-32] To account for solvation effects, the polarizable continuum model (PCM) was used with water (ε = 78.3553) as the solvent.[33] During the structural optimization process, a convergence criterion of 10^{-8} for the density matrix was implemented, and vibrational analyses were carried out to validate the results.

The free energy change of deprotonation (ΔG_a) for each species was determined by calculating the energy difference between the ionic species and its conjugate acid at their optimized geometries. The corresponding acid dissociation constant (pK_a) at 298 K were computed using standard formula:

$$pK_a = -\log\left(e^{-\frac{\Delta G_a}{RT}}\right) \quad \textit{Eq S1}$$

The Henderson-Hasselbalch equation was utilized to derive the ratio between the acidic catenane species (HX) and its conjugate base (X^-) at pH 7,[34] thereby allowing for the calculation of the percentage of the conjugate base present in the mixture.

$$pH = pKa + \log\frac{[X^-]}{[HX]} \quad \textit{Eq S2}$$

Cartesian Coordinates of the Optimized Structures

M^{+}

C	4.1267	-3.3155	-2.8045
C	3.2717	-3.9218	-3.6722
C	1.9001	-3.5123	-3.7540
C	1.4224	-2.4456	-2.9452
C	2.3314	-1.8075	-2.0116
C	3.6761	-2.2574	-1.9474
N	1.8542	-0.8181	-1.2046
C	2.6730	-0.2635	-0.3087
C	4.0280	-0.6393	-0.1995
C	4.5286	-1.6290	-1.0160
C	0.9755	-4.1371	-4.6169
C	-0.3350	-3.7105	-4.6287
C	-0.7244	-2.6381	-3.7984
N	0.1373	-2.0134	-2.9956
C	2.1155	0.7099	0.7035
O	1.2404	1.6391	0.0901
C	-2.1743	-2.2121	-3.7634
O	-2.7438	-2.8003	-2.5987
C	0.6611	2.5882	0.9049
C	-0.1661	3.5234	0.2643
C	-0.7927	4.5177	1.0046
C	-0.6113	4.6153	2.3951
C	0.2119	3.6758	3.0143
C	0.8511	2.6634	2.2873
C	-1.2651	5.7359	3.1859
C	-8.3627	-3.0765	-1.6460
N	-2.7027	5.8470	2.9294
N	-8.7574	-2.5832	-0.3244
C	-3.6235	3.0512	-4.0962
C	-4.4248	2.5225	-3.1297
C	-3.8926	1.6395	-2.1319
C	-2.5198	1.2819	-2.1644
C	-1.6741	1.8234	-3.2096
C	-2.2272	2.7267	-4.1524
N	-0.3647	1.4622	-3.2255
C	0.4444	2.0008	-4.1345
C	-0.0170	2.9238	-5.0994
C	-1.3491	3.2749	-5.1152
C	-4.6752	1.1034	-1.0883
C	-4.0811	0.3088	-0.1289
C	-2.7032	0.0298	-0.2145
N	-1.9547	0.4790	-1.2243
O	2.6422	2.7946	-3.7997
C	-1.9908	-0.7153	0.8910
O	-1.5123	-1.9573	0.3970
C	3.9870	2.6705	-3.5484
C	-0.5054	-2.5880	1.0882
C	4.7140	1.4818	-3.6838
C	6.0790	1.4715	-3.3857
C	6.7421	2.6238	-2.9539
C	5.9958	3.8038	-2.8272
C	4.6358	3.8354	-3.1204
C	0.0169	-3.7374	0.4775
C	1.0761	-4.4146	1.0696
C	1.6521	-3.9657	2.2700
C	1.0933	-2.8457	2.8845
C	0.0119	-2.1607	2.3147
C	-4.1060	-2.7817	-2.4366
C	-5.0193	-2.2624	-3.3573
C	-6.3919	-2.3448	-3.0832
C	-6.8732	-2.9261	-1.9097
C	-5.9344	-3.4147	-0.9841
C	-4.5715	-3.3473	-1.2377
C	1.9034	1.6136	-4.0945
C	2.8899	-4.6597	2.8287
C	8.2388	2.6053	-2.6871
N	3.1905	-4.3232	4.2151
N	8.6712	1.4283	-1.9293
C	4.6064	-4.0829	4.5142
C	5.1034	-2.7409	3.9580
C	6.5646	-2.4392	4.3248
C	7.0922	-1.1178	3.7340
C	7.4089	-1.1771	2.2314
C	8.2701	1.4836	-0.5189
C	8.1711	0.1064	0.1459
C	7.7419	0.1908	1.6180
C	-3.4735	4.7246	3.4723
C	-4.8122	4.5008	2.7570
C	-5.4517	3.1523	3.1192
C	-6.6521	2.7939	2.2309
C	-7.1195	1.3381	2.3872
C	-8.0416	0.8687	1.2508
C	-8.2308	-0.6562	1.1893
C	-8.7026	-1.1249	-0.1943
Cu	-0.0532	-0.1720	-1.7570
C	10.0572	1.0424	-2.1825
C	11.5963	1.7582	-0.2395
O	11.4693	0.7088	0.3465
O	12.2078	2.8008	0.3816
C	11.1681	2.0006	-1.6827
C	-3.2526	7.1724	3.1965
C	-3.8542	9.0214	4.8327
O	-4.3695	9.6540	3.9428
O	-3.8151	9.5387	6.0910
C	-3.2257	7.6456	4.6669
C	-9.9544	-3.2092	0.2254
C	-12.4604	-3.6273	0.1322
O	-12.4174	-4.1841	1.2027
O	-13.6444	-3.5381	-0.5332
C	-11.2657	-2.9894	-0.5615
C	2.4443	-5.1063	5.1940
C	3.9858	-6.8122	6.3662
O	4.3612	-6.0049	7.1827
O	4.5623	-8.0427	6.3293
C	2.8497	-6.5899	5.3731
H	5.1646	-3.6285	-2.7379
H	3.6113	-4.7298	-4.3135
H	4.6641	-0.1443	0.5266
H	5.5696	-1.9317	-0.9490
H	1.3021	-4.9544	-5.2536
H	-1.0708	-4.1882	-5.2670
H	2.9439	1.2336	1.1967
H	1.5796	0.1287	1.4703
H	-2.6816	-2.5706	-4.6673
H	-2.2601	-1.1195	-3.7233
H	-0.2938	3.4561	-0.8112
H	-1.4431	5.2318	0.5078
H	0.3681	3.7244	4.0895
H	1.4841	1.9583	2.8129
H	-0.8124	6.6925	2.8910
H	-1.0258	5.5986	4.2575
H	-8.9219	-2.5986	-2.4721
H	-8.6224	-4.1435	-1.6785
H	-4.0287	3.7312	-4.8398
H	-5.4810	2.7726	-3.0898
H	0.6846	3.3432	-5.8124
H	-1.7322	3.9729	-5.8545
H	-5.7358	1.3311	-1.0420
H	-4.6604	-0.1037	0.6896
H	-2.6671	-0.8741	1.7410
H	-1.1545	-0.0890	1.2276
H	4.2382	0.5681	-4.0226
H	6.6420	0.5473	-3.4782
H	6.4852	4.7145	-2.4886
H	4.0563	4.7470	-3.0126
H	-0.4197	-4.0709	-0.4586
H	1.4732	-5.3032	0.5822
H	1.5140	-2.5065	3.8258
H	-0.4116	-1.3121	2.8412
H	-4.6905	-1.7984	-4.2806
H	-7.0949	-1.9485	-3.8127
H	-6.2900	-3.8469	-0.0530
H	-3.8422	-3.7242	-0.5277
H	2.2189	1.2015	-5.0650
H	2.0548	0.8539	-3.3224
H	3.7510	-4.3679	2.2158
H	2.7865	-5.7495	2.6720
H	8.5242	3.5584	-2.2021
H	8.7700	2.5844	-3.6483
H	5.2577	-4.8973	4.1421
H	4.7139	-4.0806	5.6027
H	4.4546	-1.9462	4.3503
H	4.9844	-2.7247	2.8662
H	7.2101	-3.2675	3.9971
H	6.6505	-2.4025	5.4194
H	8.0035	-0.8197	4.2699
H	6.3567	-0.3217	3.9243
H	6.5576	-1.6119	1.6870
H	8.2510	-1.8654	2.0696
H	7.2803	1.9536	-0.4911
H	8.9336	2.1396	0.0762
H	9.1345	-0.4114	0.0923
H	7.4513	-0.4942	-0.4282
H	8.5439	0.6679	2.1982
H	6.8649	0.8505	1.7157
H	-3.6354	4.8107	4.5644
H	-2.8608	3.8277	3.3299
H	-4.6200	4.5355	1.6764
H	-5.5177	5.3137	2.9751
H	-5.7465	3.1413	4.1780
H	-4.6912	2.3645	3.0084
H	-6.3602	2.9549	1.1822
H	-7.4857	3.4843	2.4200
H	-7.6084	1.1942	3.3605
H	-6.2309	0.6888	2.4022
H	-7.6044	1.2032	0.2971
H	-9.0151	1.3731	1.3184
H	-8.9268	-0.9840	1.9725
H	-7.2769	-1.1592	1.3967
H	-9.6642	-0.6429	-0.4508
H	-7.9795	-0.7627	-0.9336
H	10.1667	0.9298	-3.2666
H	10.2416	0.0603	-1.7388
H	12.2453	3.5711	-0.2098
H	12.0679	1.8456	-2.2952
H	10.8782	3.0488	-1.8289
H	-4.2862	7.2038	2.8415
H	-2.7029	7.9000	2.5891
H	-3.3724	8.9194	6.6957
H	-3.7652	6.9425	5.3166
H	-2.1954	7.6832	5.0471
H	-10.0996	-2.8492	1.2481
H	-9.7766	-4.2875	0.3069
H	-13.5228	-3.0658	-1.3742
H	-11.4746	-1.9178	-0.6862
H	-11.1859	-3.4050	-1.5755
H	1.3901	-5.0729	4.8980
H	2.5240	-4.6170	6.1700
H	4.1573	-8.5839	5.6306
H	1.9887	-7.1561	5.7580
H	3.1027	-7.0483	4.4081

[M-H]

C	5.0914	-1.5967	-1.4753
C	4.5577	-2.4930	-2.3504
C	3.1403	-2.5567	-2.5635
C	2.2790	-1.6718	-1.8639
C	2.8450	-0.7393	-0.9081
C	4.2507	-0.7058	-0.7264
N	2.0023	0.0596	-0.2007
C	2.5063	0.9119	0.6961
C	3.8923	1.0249	0.9137
C	4.7606	0.2191	0.2072
C	2.5418	-3.4666	-3.4602
C	1.1729	-3.4503	-3.6338
C	0.3922	-2.5135	-2.9248
N	0.9378	-1.6543	-2.0625
C	1.5435	1.7008	1.5540
O	0.6898	2.4784	0.7340
C	-1.1011	-2.4414	-3.1521
O	-1.7561	-3.1813	-2.1293
C	-0.3135	3.1886	1.3526
C	-1.1351	3.9459	0.5023
C	-2.1853	4.6848	1.0316
C	-2.4410	4.7035	2.4138
C	-1.6080	3.9528	3.2424
C	-0.5462	3.1961	2.7301
C	-3.5574	5.5608	2.9840
C	-7.4094	-4.0152	-2.2571
N	-4.8679	5.2790	2.3893
N	-8.1575	-3.8267	-1.0145
C	-4.8987	1.4125	-1.9374
C	-5.1992	0.5345	-0.9400
C	-4.1639	-0.1329	-0.2011
C	-2.8067	0.1010	-0.5335
C	-2.4887	1.0034	-1.6224
C	-3.5362	1.6748	-2.3021
N	-1.1849	1.1642	-1.9589
C	-0.8578	2.0037	-2.9414
C	-1.8346	2.7380	-3.6459
C	-3.1684	2.5678	-3.3326
C	-4.4226	-1.0214	0.8644
C	-3.3691	-1.5882	1.5531
C	-2.0489	-1.2928	1.1674
N	-1.7827	-0.4820	0.1402
O	1.0977	3.2822	-2.5441
C	-0.8670	-1.8632	1.9158
O	-0.3732	-2.9893	1.2002
C	2.4493	3.5259	-2.5610
C	0.9215	-3.3884	1.4280
C	3.3851	2.7746	-3.2836
C	4.7368	3.1274	-3.2387
C	5.1829	4.2217	-2.4918
C	4.2318	4.9509	-1.7636
C	2.8816	4.6135	-1.7913
C	1.4156	-4.3694	0.5568
C	2.7323	-4.8013	0.6751
C	3.5939	-4.2653	1.6453
C	3.0736	-3.3187	2.5300
C	1.7459	-2.8860	2.4403
C	-3.1278	-3.2899	-2.1994
C	-3.9204	-2.7210	-3.1999
C	-5.3069	-2.9241	-3.1772
C	-5.9273	-3.6791	-2.1809
C	-5.1127	-4.2175	-1.1691
C	-3.7346	-4.0331	-1.1744
C	0.6108	2.1528	-3.2623
C	5.0681	-4.6489	1.6603
C	6.6379	4.6619	-2.5467
N	5.7141	-4.4934	2.9590
N	7.5938	3.5584	-2.4093
C	7.1215	-4.0777	2.9062
C	7.2775	-2.5825	2.5931
C	8.7401	-2.1096	2.5842
C	8.8956	-0.6047	2.2898
C	8.6886	-0.2198	0.8146
C	7.7172	3.1012	-1.0212
C	8.2676	1.6775	-0.8741
C	8.4551	1.2829	0.5997
C	-5.3980	3.9774	2.8075
C	-6.6187	3.5070	2.0062
C	-6.9431	2.0294	2.2696
C	-8.1526	1.5036	1.4804
C	-8.2841	-0.0250	1.5345
C	-9.4551	-0.6086	0.7274
C	-9.3223	-2.1207	0.4474
C	-8.4460	-2.4068	-0.7836
Cu	0.0366	-0.1470	-0.7830
C	8.8535	3.7867	-3.1147
C	10.7096	4.4862	-1.4645
O	11.1042	3.3555	-1.3000
O	11.1529	5.4746	-0.6441
C	9.7734	4.9178	-2.5877
C	-5.8027	6.3970	2.4870
C	-7.2531	7.9457	3.8856
O	-7.6437	8.4986	2.8859
O	-7.6914	8.3683	5.1031
C	-6.2820	6.7745	3.9062
C	-9.3544	-4.6980	-0.9490
C	-11.9112	-4.7178	-1.3631
O	-12.0772	-4.2685	-0.1930
O	-12.7361	-5.3012	-2.1044
C	-10.4830	-4.4644	-1.9723
C	5.3921	-5.5513	3.9121
C	7.3904	-7.1535	4.2431
O	7.9007	-6.4441	5.0774
O	8.0571	-8.2542	3.8049
C	5.9987	-6.9524	3.6527
H	6.1658	-1.5416	-1.3264
H	5.1974	-3.1699	-2.9093
H	4.2638	1.7406	1.6393
H	5.8309	0.2867	0.3664
H	3.1640	-4.1669	-4.0105
H	0.6903	-4.1382	-4.3201
H	2.1051	2.3423	2.2452
H	0.9503	0.9966	2.1572
H	-1.3410	-2.8607	-4.1382
H	-1.4220	-1.3927	-3.1337
H	-0.9183	3.9467	-0.5609
H	-2.8283	5.2575	0.3689
H	-1.7790	3.9525	4.3166
H	0.0781	2.6336	3.4147
H	-3.3336	6.6165	2.7797
H	-3.5581	5.4515	4.0854
H	-7.8543	-3.4561	-3.1037
H	-7.4965	-5.0772	-2.5229
H	-5.6889	1.9203	-2.4828
H	-6.2326	0.3278	-0.6842
H	-1.5279	3.4190	-4.4330
H	-3.9394	3.1086	-3.8744
H	-5.4461	-1.2514	1.1385
H	-3.5460	-2.2626	2.3842
H	-1.1603	-2.1551	2.9331
H	-0.0950	-1.0879	1.9819
H	3.0789	1.9306	-3.8919
H	5.4619	2.5410	-3.7956
H	4.5512	5.8031	-1.1674
H	2.1474	5.1766	-1.2239
H	0.7545	-4.7675	-0.2068
H	3.1030	-5.5593	-0.0122
H	3.7214	-2.9181	3.3037
H	1.3754	-2.1666	3.1625
H	-3.4856	-2.1312	-3.9993
H	-5.9112	-2.4899	-3.9709
H	-5.5815	-4.7908	-0.3740
H	-3.1030	-4.4575	-0.3996
H	0.7549	2.2995	-4.3420
H	1.1367	1.2418	-2.9561
H	5.5952	-4.0010	0.9495
H	5.1746	-5.6710	1.2489
H	6.7964	5.4574	-1.7941
H	6.8213	5.1301	-3.5229
H	7.7048	-4.6715	2.1765
H	7.5612	-4.2826	3.8862
H	6.7141	-2.0171	3.3475
H	6.8076	-2.3556	1.6265
H	9.3177	-2.6902	1.8499
H	9.1856	-2.3302	3.5639
H	9.8933	-0.2706	2.6040
H	8.1814	-0.0491	2.9158
H	7.8297	-0.7736	0.4072
H	9.5563	-0.5481	0.2255
H	6.7081	3.1273	-0.5925
H	8.3220	3.7992	-0.4099
H	9.2313	1.5844	-1.3845
H	7.5722	0.9888	-1.3747
H	9.2979	1.8503	1.0171
H	7.5721	1.5896	1.1835
H	-5.6365	3.9532	3.8902
H	-4.5891	3.2519	2.6634
H	-6.4098	3.6538	0.9381
H	-7.5003	4.1184	2.2418
H	-7.1115	1.8701	3.3446
H	-6.0585	1.4284	2.0122
H	-8.0611	1.8158	0.4288
H	-9.0741	1.9736	1.8529
H	-8.3641	-0.3543	2.5805
H	-7.3489	-0.4627	1.1606
H	-9.5441	-0.0719	-0.2298
H	-10.3915	-0.4121	1.2657
H	-10.3143	-2.5619	0.2933
H	-8.8906	-2.6231	1.3255
H	-8.9421	-1.9481	-1.6643
H	-7.4774	-1.8939	-0.6898
H	8.6024	4.0107	-4.1569
H	9.4346	2.8607	-3.1188
H	10.7603	6.3263	-0.8987
H	10.4225	5.2507	-3.4102
H	9.1852	5.7949	-2.2898
H	-6.6798	6.1809	1.8714
H	-5.3287	7.2758	2.0367
H	-7.2934	7.8306	5.8086
H	-6.7807	5.9239	4.3914
H	-5.4316	7.0371	4.5507
H	-9.7973	-4.5875	0.0422
H	-8.9871	-5.7311	-1.0221
H	-10.4735	-3.4124	-2.2946
H	-10.3511	-5.0702	-2.8765
H	4.3015	-5.6488	3.9273
H	5.7051	-5.2298	4.9105
H	7.5254	-8.7290	3.1438
H	5.3551	-7.7159	4.1139
H	6.0140	-7.1836	2.5795

[M-2H]$^-$

C	5.0405	-1.5568	-1.6204
C	4.5035	-2.4802	-2.4644
C	3.0870	-2.5378	-2.6833
C	2.2279	-1.6214	-2.0210
C	2.7966	-0.6651	-1.0903
C	4.2025	-0.6346	-0.9082
N	1.9569	0.1603	-0.4075
C	2.4690	1.0310	0.4664
C	3.8572	1.1505	0.6706
C	4.7219	0.3232	-0.0139
C	2.4880	-3.4720	-3.5544
C	1.1208	-3.4532	-3.7355
C	0.3425	-2.4887	-3.0614
N	0.8877	-1.5997	-2.2305
C	1.5264	1.8286	1.3384
O	0.5872	2.5387	0.5502
C	-1.1556	-2.4569	-3.2646
O	-1.7500	-3.2078	-2.2133
C	-0.3295	3.3198	1.2168
C	-1.2190	4.0446	0.4079
C	-2.1787	4.8626	0.9900
C	-2.2770	4.9925	2.3863
C	-1.3868	4.2633	3.1735
C	-0.4145	3.4289	2.6074
C	-3.2888	5.9399	3.0088
C	-7.3403	-4.3887	-2.0571
N	-4.6535	5.7240	2.5206
N	-8.0143	-3.9445	-0.8371
C	-4.5324	2.1345	-2.9025
C	-5.0535	1.3404	-1.9259
C	-4.2009	0.5780	-1.0582
C	-2.7953	0.6315	-1.2300
C	-2.2435	1.4416	-2.2974
C	-3.1145	2.2105	-3.1104
N	-0.8989	1.4315	-2.4785
C	-0.3602	2.1892	-3.4328
C	-1.1506	3.0138	-4.2623
C	-2.5216	3.0172	-4.1075
C	-4.6934	-0.2309	-0.0131
C	-3.8089	-0.8896	0.8166
C	-2.4246	-0.7597	0.5980
N	-1.9387	-0.0362	-0.4140
O	1.6830	3.2984	-2.9693
C	-1.4184	-1.4089	1.5206
O	-0.9376	-2.5990	0.9091
C	3.0542	3.3976	-2.8807
C	0.2939	-3.0802	1.2830
C	3.9556	2.4609	-3.4007
C	5.3300	2.6658	-3.2454
C	5.8358	3.7922	-2.5901
C	4.9141	4.7135	-2.0724
C	3.5408	4.5264	-2.2100
C	0.8133	-4.1020	0.4758
C	2.0719	-4.6260	0.7491
C	2.8501	-4.1443	1.8136
C	2.3021	-3.1513	2.6279
C	1.0290	-2.6249	2.3821
C	-3.1113	-3.4097	-2.2404
C	-3.9549	-3.0349	-3.2885
C	-5.3228	-3.3366	-3.2106
C	-5.8718	-3.9937	-2.1092
C	-5.0067	-4.3320	-1.0534
C	-3.6477	-4.0525	-1.1125
C	1.1411	2.1426	-3.5954
C	4.2777	-4.6392	2.0059
C	7.3369	4.0412	-2.5170
N	4.7746	-4.5138	3.3718
N	8.1101	2.8394	-2.2098
C	6.2094	-4.2217	3.4837
C	6.5397	-2.7617	3.1437
C	8.0294	-2.4207	3.3054
C	8.3664	-0.9629	2.9362
C	8.3923	-0.6791	1.4251
C	8.0582	2.5327	-0.7726
C	8.4773	1.0987	-0.4319
C	8.3848	0.8160	1.0764
C	-5.2437	4.4769	3.0164
C	-6.4053	3.9618	2.1571
C	-6.8301	2.5364	2.5421
C	-7.7899	1.8939	1.5290
C	-8.0042	0.3858	1.7520
C	-8.5073	-0.3550	0.5004
C	-8.3268	-1.8830	0.5533
C	-8.2944	-2.5097	-0.8483
Cu	0.0108	0.0411	-1.0820
C	9.4705	2.8640	-2.7876
C	11.1495	3.3501	-0.8502
O	11.4940	2.1342	-0.8509
O	11.2914	4.2073	0.0557
C	10.4802	3.8518	-2.1798
C	-5.5090	6.9042	2.6238
C	-6.7625	8.5857	4.0452
O	-6.8487	9.1627	5.2688
O	-7.3856	8.9948	3.0883
C	-5.8241	7.4001	4.0511
C	-9.1779	-4.7843	-0.4726
C	-11.5882	-5.6244	-0.8481
O	-11.7876	-5.3922	0.3744
O	-12.2623	-6.3252	-1.6421
C	-10.3323	-4.9070	-1.4740
C	4.2568	-5.5280	4.2850
C	6.0687	-7.2717	4.8712
O	6.5110	-6.6033	5.7751
O	6.7142	-8.4115	4.5061
C	4.7803	-6.9754	4.1110
H	6.1142	-1.5015	-1.4667
H	5.1393	-3.1833	-2.9948
H	4.2348	1.8914	1.3670
H	5.7938	0.4015	0.1279
H	3.1091	-4.1942	-4.0770
H	0.6364	-4.1628	-4.3982
H	2.1073	2.5225	1.9587
H	1.0037	1.1338	2.0148
H	-1.3990	-2.8986	-4.2398
H	-1.5179	-1.4214	-3.2506
H	-1.1277	3.9607	-0.6704
H	-2.8737	5.4114	0.3607
H	-1.4405	4.3420	4.2571
H	0.2562	2.8842	3.2616
H	-3.0158	6.9717	2.7488
H	-3.2097	5.8690	4.1101
H	-7.8396	-4.0401	-2.9810
H	-7.4076	-5.4846	-2.0765
H	-5.1835	2.7192	-3.5460
H	-6.1279	1.2780	-1.7804
H	-0.6710	3.6259	-5.0190
H	-3.1517	3.6292	-4.7469
H	-5.7628	-0.3239	0.1344
H	-4.1669	-1.5070	1.6334
H	-1.8783	-1.6353	2.4919
H	-0.5951	-0.7023	1.6786
H	3.6080	1.5800	-3.9296
H	6.0337	1.9333	-3.6301
H	5.2779	5.5961	-1.5500
H	2.8299	5.2390	-1.8026
H	0.2197	-4.4528	-0.3624
H	2.4654	-5.4151	0.1110
H	2.8837	-2.7898	3.4703
H	0.6287	-1.8726	3.0535
H	-3.5747	-2.5207	-4.1645
H	-5.9685	-3.0525	-4.0391
H	-5.4230	-4.8155	-0.1738
H	-2.9777	-4.3120	-0.2988
H	1.4075	2.1283	-4.6626
H	1.5211	1.2285	-3.1278
H	4.9346	-4.0452	1.3597
H	4.3517	-5.6731	1.6166
H	7.5268	4.8712	-1.8101
H	7.6716	4.4040	-3.4983
H	6.8223	-4.8950	2.8533
H	6.5039	-4.4215	4.5180
H	5.9418	-2.1147	3.7996
H	6.2199	-2.5362	2.1178
H	8.6372	-3.1033	2.6929
H	8.3211	-2.6072	4.3484
H	9.3426	-0.6939	3.3616
H	7.6343	-0.2987	3.4201
H	7.5245	-1.1574	0.9459
H	9.2769	-1.1570	0.9807
H	7.0146	2.6788	-0.4583
H	8.6688	3.2404	-0.1865
H	9.5135	0.9433	-0.7483
H	7.8369	0.4031	-0.9982
H	9.2224	1.3203	1.5755
H	7.4689	1.2656	1.4967
H	-5.5604	4.5521	4.0752
H	-4.4471	3.7248	2.9938
H	-6.0759	3.9762	1.1095
H	-7.2719	4.6332	2.2213
H	-7.2772	2.5303	3.5463
H	-5.9286	1.9088	2.6078
H	-7.3662	2.0412	0.5240
H	-8.7531	2.4226	1.5259
H	-8.6841	0.2143	2.5975
H	-7.0437	-0.0577	2.0541
H	-7.9512	0.0299	-0.3699
H	-9.5574	-0.1001	0.3036
H	-9.1161	-2.3389	1.1626
H	-7.3768	-2.1313	1.0474
H	-9.2303	-2.2708	-1.3878
H	-7.4869	-2.0223	-1.4100
H	9.3349	3.0535	-3.8622
H	9.9075	1.8660	-2.6865
H	11.2905	3.9815	-2.9146
H	10.0361	4.8426	-2.0222
H	-6.4517	6.7030	2.1081
H	-5.0293	7.7180	2.0674
H	-6.2991	6.6122	4.6507
H	-4.9162	7.6823	4.5967
H	-9.6039	-4.4111	0.4624
H	-8.7852	-5.7861	-0.2476
H	-10.6554	-3.9037	-1.7941
H	-10.0319	-5.4443	-2.3817
H	3.1682	-5.5415	4.1672
H	4.4708	-5.2163	5.3124
H	4.0282	-7.6801	4.4952
H	4.9081	-7.2237	3.0493
H	6.2432	-8.8449	3.7745
H	-7.4814	9.9010	5.1787

[M-3H]$^{2-}$

C	-5.0646	1.4901	-1.5483
C	-4.5202	2.3800	-2.4229
C	-3.1062	2.4007	-2.6627
C	-2.2575	1.4829	-1.9886
C	-2.8329	0.5634	-1.0257
C	-4.2364	0.5682	-0.8244
N	-2.0010	-0.2620	-0.3334
C	-2.5192	-1.0979	0.5705
C	-3.9072	-1.1825	0.7952
C	-4.7638	-0.3555	0.1008
C	-2.5002	3.2986	-3.5663
C	-1.1369	3.2442	-3.7669
C	-0.3699	2.2802	-3.0792
N	-0.9213	1.4261	-2.2164
C	-1.5840	-1.8880	1.4572
O	-0.6498	-2.6196	0.6872
C	1.1234	2.2106	-3.3052
O	1.7510	2.9694	-2.2796
C	0.2878	-3.3608	1.3778
C	1.1950	-4.0859	0.5906
C	2.1839	-4.8508	1.1964
C	2.2948	-4.9293	2.5954
C	1.3796	-4.2071	3.3599
C	0.3771	-3.4246	2.7706
C	3.3610	-5.8043	3.2368
C	7.3291	4.2102	-2.3447
N	4.6909	-5.5780	2.6788
N	8.0428	3.8359	-1.1253
C	4.3852	-2.5479	-2.8110
C	4.9429	-1.7686	-1.8429
C	4.1282	-0.9416	-0.9985
C	2.7232	-0.9157	-1.1831
C	2.1340	-1.7171	-2.2372
C	2.9669	-2.5481	-3.0289
N	0.7915	-1.6454	-2.4218
C	0.2178	-2.3966	-3.3604
C	0.9679	-3.2757	-4.1713
C	2.3364	-3.3441	-4.0116
C	4.6589	-0.1461	0.0378
C	3.8074	0.5807	0.8450
C	2.4198	0.5275	0.6158
N	1.9001	-0.1856	-0.3868
O	-1.8723	-3.4347	-2.9404
C	1.4454	1.2392	1.5264
O	0.9895	2.4224	0.8830
C	-3.2462	-3.4765	-2.8521
C	-0.2236	2.9483	1.2562
C	-4.1069	-2.4882	-3.3448
C	-5.4888	-2.6366	-3.1904
C	-6.0420	-3.7558	-2.5616
C	-5.1604	-4.7302	-2.0719
C	-3.7808	-4.6004	-2.2100
C	-0.7225	3.9632	0.4274
C	-1.9636	4.5288	0.6981
C	-2.7449	4.0962	1.7815
C	-2.2164	3.1103	2.6166
C	-0.9601	2.5432	2.3738
C	3.1096	3.1783	-2.3559
C	3.9172	2.8052	-3.4327
C	5.2831	3.1229	-3.4088
C	5.8653	3.7947	-2.3337
C	5.0383	4.1270	-1.2462
C	3.6816	3.8301	-1.2512
C	-1.2796	-2.2829	-3.5271
C	-4.1569	4.6349	1.9718
C	-7.5522	-3.9423	-2.4875
N	-4.6511	4.5468	3.3418
N	-8.2758	-2.7120	-2.1733
C	-6.0936	4.3007	3.4647
C	-6.4715	2.8473	3.1461
C	-7.9703	2.5558	3.3222
C	-8.3586	1.1076	2.9654
C	-8.4053	0.8164	1.4564
C	-8.2082	-2.4112	-0.7354
C	-8.5648	-0.9605	-0.3943
C	-8.4649	-0.6791	1.1138
C	5.2288	-4.2793	3.0889
C	6.5288	-3.8931	2.3725
C	6.8985	-2.4218	2.6209
C	7.9200	-1.8597	1.6202
C	8.1023	-0.3322	1.7191
C	8.5510	0.3268	0.4034
C	8.3750	1.8546	0.3717
C	8.3263	2.4024	-1.0617
Cu	-0.0576	-0.2007	-1.0404
C	-9.6371	-2.6802	-2.7485
C	-11.3280	-3.1189	-0.8089
O	-11.6137	-1.8879	-0.7903
O	-11.5125	-3.9825	0.0830
C	-10.6831	-3.6324	-2.1460
C	5.6185	-6.7097	2.8885
C	7.6015	-7.4203	4.4065
O	7.9013	-8.2764	5.2723
O	8.3627	-6.7978	3.6130
C	6.0777	-7.0303	4.3242
C	9.2122	4.6978	-0.8427
C	11.5961	5.5456	-1.3481
O	11.8364	5.3983	-0.1198
O	12.2340	6.2026	-2.2069
C	10.3342	4.7702	-1.8854
C	-4.0989	5.5598	4.2360
C	-5.8545	7.3683	4.7968
O	-6.3131	6.7310	5.7149
O	-6.4676	8.5192	4.4111
C	-4.5778	7.0196	4.0391
H	-6.1369	1.4616	-1.3787
H	-5.1482	3.0831	-2.9625
H	-4.2902	-1.8967	1.5162
H	-5.8351	-0.4073	0.2581
H	-3.1133	4.0209	-4.0983
H	-0.6470	3.9253	-4.4550
H	-2.1719	-2.5633	2.0921
H	-1.0589	-1.1828	2.1214
H	1.3578	2.6278	-4.2930
H	1.4647	1.1682	-3.2761
H	1.1003	-4.0392	-0.4898
H	2.9035	-5.3908	0.5878
H	1.4423	-4.2452	4.4454
H	-0.3101	-2.8815	3.4092
H	3.1107	-6.8587	3.0550
H	3.3227	-5.6670	4.3360
H	7.8053	3.8227	-3.2655
H	7.3810	5.3048	-2.4204
H	5.0065	-3.1821	-3.4369
H	6.0172	-1.7691	-1.6836
H	0.4594	-3.8798	-4.9154
H	2.9365	-4.0013	-4.6349
H	5.7306	-0.1208	0.1990
H	4.1938	1.1881	1.6566
H	1.9243	1.4838	2.4840
H	0.6045	0.5624	1.7184
H	-3.7219	-1.6095	-3.8511
H	-6.1609	-1.8647	-3.5538
H	-5.5613	-5.6089	-1.5705
H	-3.1009	-5.3538	-1.8236
H	-0.1282	4.2746	-0.4257
H	-2.3414	5.3116	0.0429
H	-2.8001	2.7859	3.4726
H	-0.5744	1.7981	3.0614
H	3.5108	2.2787	-4.2894
H	5.9006	2.8389	-4.2585
H	5.4843	4.6180	-0.3858
H	3.0403	4.0842	-0.4130
H	-1.5398	-2.2249	-4.5946
H	-1.6241	-1.3690	-3.0332
H	-4.8336	4.0492	1.3385
H	-4.2035	5.6638	1.5656
H	-7.7753	-4.7676	-1.7847
H	-7.9029	-4.2863	-3.4701
H	-6.6889	4.9841	2.8283
H	-6.3762	4.5236	4.4976
H	-5.8894	2.1902	3.8060
H	-6.1665	2.5993	2.1210
H	-8.5605	3.2538	2.7099
H	-8.2471	2.7587	4.3662
H	-9.3403	0.8746	3.3995
H	-7.6459	0.4219	3.4482
H	-7.5201	1.2543	0.9704
H	-9.2706	1.3302	1.0140
H	-7.1714	-2.6021	-0.4231
H	-8.8477	-3.0932	-0.1495
H	-9.5922	-0.7603	-0.7135
H	-7.8925	-0.2947	-0.9590
H	-9.3267	-1.1426	1.6110
H	-7.5724	-1.1693	1.5390
H	5.3860	-4.2239	4.1853
H	4.4620	-3.5289	2.8559
H	6.3767	-4.0516	1.2947
H	7.3435	-4.5575	2.6870
H	7.2658	-2.2897	3.6489
H	5.9859	-1.8094	2.5478
H	7.5676	-2.1068	0.6067
H	8.8856	-2.3719	1.7287
H	8.7987	-0.0782	2.5299
H	7.1399	0.1141	2.0131
H	7.9545	-0.1070	-0.4157
H	9.5913	0.0553	0.1767
H	9.1728	2.3424	0.9451
H	7.4306	2.1299	0.8616
H	9.2570	2.1376	-1.5978
H	7.5140	1.8830	-1.5859
H	-9.5107	-2.8675	-3.8248
H	-10.0349	-1.6667	-2.6400
H	-11.5004	-3.7231	-2.8789
H	-10.2785	-4.6417	-1.9995
H	6.5245	-6.5061	2.3136
H	5.1392	-7.5904	2.4378
H	5.9609	-6.1390	4.9584
H	5.4725	-7.8186	4.7874
H	9.6677	4.3778	0.0980
H	8.8218	5.7095	-0.6610
H	10.6626	3.7524	-2.1492
H	9.9975	5.2423	-2.8164
H	-3.0108	5.5377	4.1146
H	-4.3187	5.2723	5.2692
H	-3.8033	7.7068	4.4105
H	-4.7000	7.2543	2.9737
H	-5.9865	8.9242	3.6698

[M-4H]$^{3-}$

C	5.0551	-1.5652	-1.5007
C	4.5123	-2.4406	-2.3907
C	3.1002	-2.4500	-2.6417
C	2.2516	-1.5355	-1.9633
C	2.8253	-0.6297	-0.9864
C	4.2269	-0.6463	-0.7732
N	1.9932	0.1940	-0.2929
C	2.5092	1.0168	0.6241
C	3.8952	1.0875	0.8637
C	4.7522	0.2619	0.1681
C	2.4956	-3.3348	-3.5590
C	1.1336	-3.2724	-3.7668
C	0.3668	-2.3131	-3.0725
N	0.9172	-1.4702	-2.1984
C	1.5700	1.8092	1.5043
O	0.6702	2.5745	0.7246
C	-1.1255	-2.2363	-3.3030
O	-1.7594	-2.9922	-2.2792
C	-0.2711	3.3199	1.4042
C	-1.1537	4.0648	0.6071
C	-2.1434	4.8373	1.2017
C	-2.2794	4.9049	2.5991
C	-1.3889	4.1627	3.3736
C	-0.3866	3.3716	2.7956
C	-3.3445	5.7897	3.2286
C	-7.3440	-4.2004	-2.3473
N	-4.6709	5.5755	2.6571
N	-8.0598	-3.8151	-1.1322
C	-4.3987	2.5086	-2.7270
C	-4.9521	1.7197	-1.7640
C	-4.1341	0.8842	-0.9315
C	-2.7303	0.8587	-1.1237
C	-2.1462	1.6695	-2.1730
C	-2.9815	2.5099	-2.9516
N	-0.8046	1.5983	-2.3646
C	-0.2345	2.3587	-3.2974
C	-0.9867	3.2488	-4.0948
C	-2.3542	3.3167	-3.9280
C	-4.6598	0.0796	0.1005
C	-3.8042	-0.6544	0.8969
C	-2.4175	-0.6006	0.6611
N	-1.9032	0.1207	-0.3385
O	1.8580	3.4335	-2.9900
C	-1.4387	-1.3194	1.5617
O	-0.9696	-2.4850	0.9014
C	3.2325	3.4737	-2.9064
C	0.2503	-3.0056	1.2764
C	4.0867	2.4534	-3.3411
C	5.4697	2.6022	-3.1976
C	6.0300	3.7517	-2.6340
C	5.1548	4.7589	-2.2018
C	3.7746	4.6304	-2.3324
C	0.7632	-4.0049	0.4384
C	2.0079	-4.5632	0.7116
C	2.7797	-4.1397	1.8054
C	2.2360	-3.1680	2.6471
C	0.9766	-2.6071	2.4025
C	-3.1185	-3.1929	-2.3565
C	-3.9236	-2.8149	-3.4334
C	-5.2913	-3.1248	-3.4099
C	-5.8779	-3.7935	-2.3352
C	-5.0529	-4.1310	-1.2476
C	-3.6946	-3.8420	-1.2521
C	1.2606	2.2387	-3.4769
C	4.1918	-4.6779	2.0092
C	7.5412	3.9348	-2.5690
N	4.7013	-4.5266	3.3634
N	8.2641	2.7066	-2.2481
C	6.1499	-4.2949	3.4334
C	6.5139	-2.8280	3.1606
C	8.0193	-2.5388	3.2720
C	8.4014	-1.0891	2.9133
C	8.4262	-0.8004	1.4033
C	8.2052	2.4161	-0.8073
C	8.5564	0.9658	-0.4594
C	8.4713	0.6934	1.0510
C	-5.2253	4.2827	3.0641
C	-6.5257	3.9102	2.3412
C	-6.9152	2.4446	2.5928
C	-7.9397	1.8924	1.5894
C	-8.1442	0.3674	1.6940
C	-8.5654	-0.2947	0.3707
C	-8.4052	-1.8244	0.3506
C	-8.3339	-2.3795	-1.0790
Cu	0.0499	0.1345	-1.0021
C	9.6207	2.6665	-2.8332
C	11.3311	3.1251	-0.9150
O	11.6177	1.8947	-0.8877
O	11.5254	3.9979	-0.0340
C	10.6719	3.6253	-2.2502
C	-5.5894	6.7164	2.8566
C	-7.5769	7.4610	4.3511
O	-7.8710	8.3274	5.2085
O	-8.3402	6.8474	3.5527
C	-6.0597	7.0432	4.2873
C	-9.2367	-4.6677	-0.8517
C	-11.6271	-5.4954	-1.3619
O	-11.8698	-5.3417	-0.1349
O	-12.2690	-6.1486	-2.2207
C	-10.3557	-4.7345	-1.8980
C	4.1949	-5.5509	4.2991
C	6.0883	-7.2640	4.9514
O	6.4053	-6.4322	5.8474
O	6.6714	-8.3262	4.6257
C	4.7580	-6.9748	4.1610
H	6.1255	-1.5480	-1.3192
H	5.1400	-3.1425	-2.9322
H	4.2765	1.7890	1.5981
H	5.8221	0.3015	0.3387
H	3.1086	-4.0544	-4.0947
H	0.6447	-3.9444	-4.4645
H	2.1565	2.4608	2.1646
H	1.0137	1.1041	2.1422
H	-1.3595	-2.6521	-4.2917
H	-1.4623	-1.1923	-3.2743
H	-1.0396	4.0264	-0.4717
H	-2.8440	5.3925	0.5845
H	-1.4715	4.1918	4.4580
H	0.2807	2.8124	3.4415
H	-3.0828	6.8416	3.0492
H	-3.3193	5.6523	4.3282
H	-7.8155	-3.8151	-3.2715
H	-7.4024	-5.2951	-2.4170
H	-5.0226	3.1496	-3.3432
H	-6.0256	1.7193	-1.5997
H	-0.4798	3.8614	-4.8330
H	-2.9565	3.9820	-4.5407
H	-5.7305	0.0528	0.2670
H	-4.1864	-1.2686	1.7052
H	-1.9183	-1.5817	2.5147
H	-0.6056	-0.6367	1.7685
H	3.6958	1.5486	-3.7941
H	6.1378	1.8075	-3.5163
H	5.5616	5.6622	-1.7510
H	3.0996	5.4087	-1.9892
H	0.1765	-4.3114	-0.4221
H	2.3967	-5.3364	0.0511
H	2.8178	-2.8566	3.5092
H	0.5797	-1.8734	3.0967
H	-3.5139	-2.2904	-4.2897
H	-5.9071	-2.8371	-4.2597
H	-5.5017	-4.6200	-0.3874
H	-3.0539	-4.0994	-0.4142
H	1.5055	2.0996	-4.5410
H	1.6158	1.3663	-2.9202
H	4.8572	-4.1229	1.3340
H	4.2256	-5.7266	1.6559
H	7.7696	4.7663	-1.8751
H	7.8873	4.2699	-3.5565
H	6.7050	-4.9448	2.7300
H	6.4782	-4.5942	4.4336
H	5.9675	-2.1971	3.8755
H	6.1535	-2.5351	2.1635
H	8.5804	-3.2323	2.6273
H	8.3408	-2.7571	4.2997
H	9.3889	-0.8504	3.3323
H	7.6929	-0.4044	3.4043
H	7.5384	-1.2499	0.9331
H	9.2893	-1.3096	0.9511
H	7.1717	2.6135	-0.4887
H	8.8526	3.0992	-0.2314
H	9.5791	0.7565	-0.7880
H	7.8736	0.3006	-1.0119
H	9.3341	1.1659	1.5381
H	7.5793	1.1812	1.4803
H	-5.3890	4.2292	4.1597
H	-4.4654	3.5240	2.8353
H	-6.3655	4.0629	1.2638
H	-7.3343	4.5856	2.6483
H	-7.2887	2.3206	3.6196
H	-6.0104	1.8203	2.5257
H	-7.5783	2.1301	0.5770
H	-8.8982	2.4191	1.6907
H	-8.8657	0.1277	2.4869
H	-7.1962	-0.0891	2.0173
H	-7.9429	0.1280	-0.4347
H	-9.5964	-0.0135	0.1152
H	-9.2195	-2.3003	0.9106
H	-7.4737	-2.1071	0.8608
H	-9.2524	-2.1095	-1.6335
H	-7.5078	-1.8690	-1.5903
H	9.4863	2.8411	-3.9107
H	10.0190	1.6544	-2.7155
H	11.4819	3.7097	-2.9922
H	10.2680	4.6356	-2.1093
H	-6.4916	6.5216	2.2727
H	-5.0971	7.5922	2.4102
H	-5.9673	6.1484	4.9208
H	-5.4456	7.8193	4.7591
H	-9.6932	-4.3423	0.0867
H	-8.8542	-5.6816	-0.6663
H	-10.6739	-3.7149	-2.1675
H	-10.0204	-5.2138	-2.8257
H	3.1017	-5.5498	4.1882
H	4.4331	-5.2170	5.3142
H	4.0093	-7.6848	4.5466
H	4.9086	-7.2585	3.1111

6. Supplementary References

(1) Yee, C.-C.; Ng, A. W. H.; Au-Yeung, H. Y., Control over the macrocyclisation pathway and product topology in a copper-templated catenane synthesis. *Chem. Commun.* **2019,** *55*, 6169-6172.

(2) Zhu, L.; Li, J.; Yang, J.; Au-Yeung, H. Y., Cross dehydrogenative C–O coupling catalysed by a catenane-coordinated copper(I). *Chem. Sci.* **2020,** *11*, 13008-13014.

(3) Tang, M. P.; Zhu, L.; Deng, Y.; Shi, Y.-X.; Kin-Man Lai, S.; Mo, X.; Pang, X.-Y.; Liu, C.; Jiang, W.; Tse, E. C. M.; Au-Yeung, H. Y., Water and air stable copper(I) complexes of tetracationic catenane ligands for oxidative C−C cross-coupling. *Angew. Chem. Int. Ed.* **2024,** *63*, e202405971.

(4) Chen, Y.; Zhang, P.; Jiao, L.; Chen, G.; Yang, Y.; Chong, H.; Lin, M., High efficient and selective removal of U(VI) from lanthanides by phenanthroline diamide functionalized carbon doped boron nitride. *J. Chem. Eng.* **2022,** *446*, 137337.

(5) Ravi, S.; Zhang, S.; Lee, Y.-R.; Kang, K.-K.; Kim, J.-M.; Ahn, J.-W.; Ahn, W.-S., EDTA-functionalized KCC-1 and KIT-6 mesoporous silicas for Nd^{3+} ion recovery from aqueous solutions. *J. Ind. Eng. Chem.* **2018,** *67*, 210-218.

(6) Shi, L.; Li, Y.; He, S.; Liu, Y.; Tang, X.; Ao, L.; Lv, X.; Fu, W.; Jiang, G., Efficient electrocatalytic nitrate reduction on molecular catalyst with electron-deficient single-atom $Cu^{\delta+}$ sites. *J. Chem. Eng.* **2024,** *495*, 153427.

(7) Jiang, Z.; Wang, Y.; Lin, Z.; Yuan, Y.; Zhang, X.; Tang, Y.; Wang, H.; Li, H.; Jin, C.; Liang, Y., Molecular electrocatalysts for rapid and selective reduction of nitrogenous waste to ammonia. *Energy Environ. Sci.* **2023,** *16*, 2239-2246.

(8) Chebotareva, N.; Nyokong, T., Metallophthalocyanine catalysed electroreduction of nitrate and nitrite ions in alkaline media. *J. Appl. Electrochem.* **1997,** *27*, 975-981.

(9) Sun, L.; Dai, C.; Wang, T.; Jin, X.; Xu, Z. J.; Wang, X., Modulating the electronic structure of cobalt in molecular catalysts via coordination environment regulation for highly efficient heterogeneous nitrate reduction. *Angew. Chem. Int. Ed.* **2024,** *63*, e202320027.

(10) Xue, Y.; Yu, Q.; Ma, Q.; Chen, Y.; Zhang, C.; Teng, W.; Fan, J.; Zhang, W.-x., Electrocatalytic hydrogenation boosts reduction of nitrate to ammonia over single-atom Cu with Cu(I)-N_3C_1 sites. *Environ. Sci. Technol.* **2022,** *56*, 14797-14807.

(11) Yang, J.; Qi, H.; Li, A.; Liu, X.; Yang, X.; Zhang, S.; Zhao, Q.; Jiang, Q.; Su, Y.; Zhang, L.; Li, J.-F.; Tian, Z.-Q.; Liu, W.; Wang, A.; Zhang, T., Potential-driven

restructuring of Cu single atoms to nanoparticles for boosting the electrochemical reduction of nitrate to ammonia. *J. Am. Chem. Soc.* **2022,** *144*, 12062-12071.
(12) Chen, H.; Zhang, C.; Sheng, L.; Wang, M.; Fu, W.; Gao, S.; Zhang, Z.; Chen, S.; Si, R.; Wang, L.; Yang, B., Copper single-atom catalyst as a high-performance electrocatalyst for nitrate-ammonium conversion. *J. Hazard. Mater.* **2022,** *434*, 128892.
(13) Cheng, X.-F.; He, J.-H.; Ji, H.-Q.; Zhang, H.-Y.; Cao, Q.; Sun, W.-J.; Yan, C.-L.; Lu, J.-M., Coordination symmetry breaking of single-atom catalysts for robust and efficient nitrate electroreduction to ammonia. *Adv. Mater.* **2022,** *34*, 2205767.
(14) Cheng, J.; Sun, W.; Dai, G.; Yang, X.; Xia, R.; Xu, Y.; Yang, X.; Tu, W., Electroreduction of nitrate to ammonia on atomically-dispersed Cu-N_4 active sites with high efficiency and stability. *Fuel* **2023,** *332*, 126106.
(15) Pletcher, D.; Poorabedi, Z., The reduction of nitrate at a copper cathode in aqueous acid. *Electrochim. Acta* **1979,** *24*, 1253-1256.
(16) Wu, K.; Sun, C.; Wang, Z.; Song, Q.; Bai, X.; Yu, X.; Li, Q.; Wang, Z.; Zhang, H.; Zhang, J.; Tong, X.; Liang, Y.; Khosla, A.; Zhao, Z., Surface reconstruction on uniform Cu nanodisks boosted electrochemical nitrate reduction to ammonia. *ACS Materials Lett.* **2022,** *4*, 650-656.
(17) Fu, X.; Zhao, X.; Hu, X.; He, K.; Yu, Y.; Li, T.; Tu, Q.; Qian, X.; Yue, Q.; Wasielewski, M. R.; Kang, Y., Alternative route for electrochemical ammonia synthesis by reduction of nitrate on copper nanosheets. *Appl. Mater. Today* **2020,** *19*, 100620.
(18) Ma, G.; Sun, F.; Qiao, L.; Shen, Q.; Wang, L.; Tang, Q.; Tang, Z., Atomically precise alkynyl-protected $Ag_{20}Cu_{12}$ nanocluster: Structure analysis and electrocatalytic performance toward nitrate reduction for NH_3 synthesis. *Nano Res.* **2023,** *16*, 10867-10872.
(19) Ji, X.-Y.; Sun, K.; Liu, Z.-K.; Liu, X.; Dong, W.; Zuo, X.; Shao, R.; Tao, J., Identification of dynamic active sites among Cu species derived from MOFs@CuPc for electrocatalytic nitrate reduction reaction to ammonia. *Nano-Micro Lett.* **2023,** *15*, 110.
(20) Xu, Y.-T.; Xie, M.-Y.; Zhong, H.; Cao, Y., In situ clustering of single-atom copper precatalysts in a metal-organic framework for efficient electrocatalytic nitrate-to-ammonia reduction. *ACS Catal.* **2022,** *12*, 8698-8706.
(21) Wang, B.; Ma, J.; Yang, R.; Meng, B.; Yang, X.; Zhang, Q.; Zhang, B.; Zhuo, S., Bridging nickel-MOF and copper single atoms/clusters with H-substituted graphdiyne for the tandem catalysis of nitrate to ammonia. *Angew. Chem. Int.*

Ed. **2024,** *63*, e202404819.
(22) Ren, T.; Ren, K.; Wang, M.; Liu, M.; Wang, Z.; Wang, H.; Li, X.; Wang, L.; Xu, Y., Concave-convex surface oxide layers over copper nanowires boost electrochemical nitrate-to-ammonia conversion. *Chem. Eng. J.* **2021,** *426*, 130759.
(23) Gong, Z.; Zhong, W.; He, Z.; Liu, Q.; Chen, H.; Zhou, D.; Zhang, N.; Kang, X.; Chen, Y., Regulating surface oxygen species on copper (I) oxides via plasma treatment for effective reduction of nitrate to ammonia. *Appl. Catal. B Environ.* **2022,** *305*, 121021.
(24) Wang, C.; Ye, F.; Shen, J.; Xue, K.-H.; Zhu, Y.; Li, C., In situ loading of Cu_2O active sites on island-like copper for efficient electrochemical reduction of nitrate to ammonia. *ACS Appl. Mater. Interfaces* **2022,** *14*, 6680-6688.
(25) Qin, J.; Chen, L.; Wu, K.; Wang, X.; Zhao, Q.; Li, L.; Liu, B.; Ye, Z., Electrochemical synthesis of ammonium from nitrates via surface engineering in Cu_2O(100) facets. *ACS Appl. Energy Mater.* **2022,** *5*, 71-76.
(26) Hanwell, M. D.; Curtis, D. E.; Lonie, D. C.; Vandermeersch, T.; Zurek, E.; Hutchison, G. R., Avogadro: an advanced semantic chemical editor, visualization, and analysis platform. *J. Cheminform.* **2012,** *4*, 17.
(27) Frisch, M.; Trucks, G.; Schlegel, H. B.; Scuseria, G. E.; Robb, M. A.; Cheeseman, J. R.; Scalmani, G.; Barone, V.; Mennucci, B.; Petersson, G., gaussian 09, Gaussian. *Inc., Wallingford CT* **2009,** *121*, 150-166.
(28) Becke, A. D., Density‐functional thermochemistry. III. The role of exact exchange. *J. Chem. Phys.* **1993,** *98*, 5648-5652.
(29) Lee, C.; Yang, W.; Parr, R. G., Development of the Colle-Salvetti correlation-energy formula into a functional of the electron density. *Phys. Rev. B* **1988,** *37*, 785-789.
(30) Stephens, P. J.; Devlin, F. J.; Chabalowski, C. F.; Frisch, M. J., Ab initio calculation of vibrational absorption and circular dichroism spectra using density functional force fields. *J. Phys. Chem.* **1994,** *98*, 11623-11627.
(31) Ben El Ayouchia, H.; Bahsis, L.; Anane, H.; Domingo, L. R.; Stiriba, S.-E., Understanding the mechanism and regioselectivity of the copper(I) catalyzed [3 + 2] cycloaddition reaction between azide and alkyne: a systematic DFT study. *RSC Adv.* **2018,** *8*, 7670-7678.
(32) Yang, Y.; Weaver, M. N.; Merz, K. M., Jr., Assessment of the "6-31+G** + LANL2DZ" mixed basis set coupled with density functional theory methods and the effective core potential: prediction of heats of formation and ionization potentials for first-row-transition-metal complexes. *J. Phys. Chem. A* **2009,** *113*, 9843-9851.

(33) Tomasi, J.; Mennucci, B.; Cammi, R., Quantum mechanical continuum solvation models. *Chem. Rev.* **2005,** *105*, 2999-3094.
(34) Po, H. N.; Senozan, N. M., The Henderson-Hasselbalch equation: its history and limitations. *J. Chem. Educ.* **2001,** *78*, 1499.
